# Deep learning in drug discovery: an integrative review and future challenges

**DOI:** 10.1007/s10462-022-10306-1

**Published:** 2022-11-17

**Authors:** Heba Askr, Enas Elgeldawi, Heba Aboul Ella, Yaseen A. M. M. Elshaier, Mamdouh M. Gomaa, Aboul Ella Hassanien

**Affiliations:** 1 Faculty of Computers and Artificial Intelligence, University of Sadat City, Sadat City, Egypt; 2 Computer Science Department, Faculty of Science, Minia University, Minia, Egypt; 3 Faculty of Computers and Artificial Intelligence, Cairo University, Cairo, Egypt; 4 Faculty of Pharmacy and Drug Technology, Chinese University in Egypt (CUE), Cairo, Egypt; 5 Faculty of Pharmacy, University of Sadat City, Sadat City, Menoufia Egypt

**Keywords:** Drug discovery, Artificial intelligence, Deep learning, Drug–target interactions, Drug–drug similarity, Drug side-effects, Drug sensitivity and response, Drug dosing optimization, Explainable artificial intelligence, Digital twining

## Abstract

Recently, using artificial intelligence (AI) in drug discovery has received much attention since it significantly shortens the time and cost of developing new drugs. Deep learning (DL)-based approaches are increasingly being used in all stages of drug development as DL technology advances, and drug-related data grows. Therefore, this paper presents a systematic Literature review (SLR) that integrates the recent DL technologies and applications in drug discovery Including, drug–target interactions (DTIs), drug–drug similarity interactions (DDIs), drug sensitivity and responsiveness, and drug-side effect predictions. We present a review of more than 300 articles between 2000 and 2022. The benchmark data sets, the databases, and the evaluation measures are also presented. In addition, this paper provides an overview of how explainable AI (XAI) supports drug discovery problems. The drug dosing optimization and success stories are discussed as well. Finally, digital twining (DT) and open issues are suggested as future research challenges for drug discovery problems. Challenges to be addressed, future research directions are identified, and an extensive bibliography is also included.

## Introduction

The examination of how various drugs interact with the body and how a medication needs to act on the body to have a therapeutic impact is known as drug discovery. Drug discovery strategy constitutes from different approaches as physiology-based and target based. This strategy is based on information about the ligand and the target. In this regard, our attention was directed in certain topics especially drug (ligand)–target interactions, drug sensitivity and response, drug–drug interaction, and drug–drug similarity. For certain diseases such as cancer or pandemic situations as COVID-19, more than one drug combination is required to alleviate the prognosis and pathogenesis interactions. Despite all the recent advances in pharmaceuticals, medication development is still a labor-intensive and costly process. As a result, several computational algorithms are proposed to speed up the drug discovery process (Betsabeh and Mansoor 2021).

As DL models progress and the drug data size is getting bigger, a slew of new DL-based approaches is cropping up at every stage of the drug development process (Kim et al. 2021). In addition, we’ve seen large pharmaceutical corporations migrate toward AI in the wake of the development of DL approaches, eschewing outmoded, ineffective procedures to increase patient profit while also increasing their own (Nag et al. 2022). Despite the DL impressive performance, it remains a critical and challenging task, and there is a chance for researchers to develop several algorithms that improve drug discovery performance. Therefore, this paper presents a SLR that integrates the recent DL technologies and applications in drug discovery. This review study is the first one that incorporates the recent DL models and applications for the different categories of drug discovery problems such as DTIs, DDIs similarity, drug sensitivity and response, and drug-side effects predictions, as well as presenting new challenging topics such as XAI and DT and how they help the advancement of the drug discovery problems. In addition, the paper supports the researchers with the most frequently used datasets in the field.

**Fig. 1:**
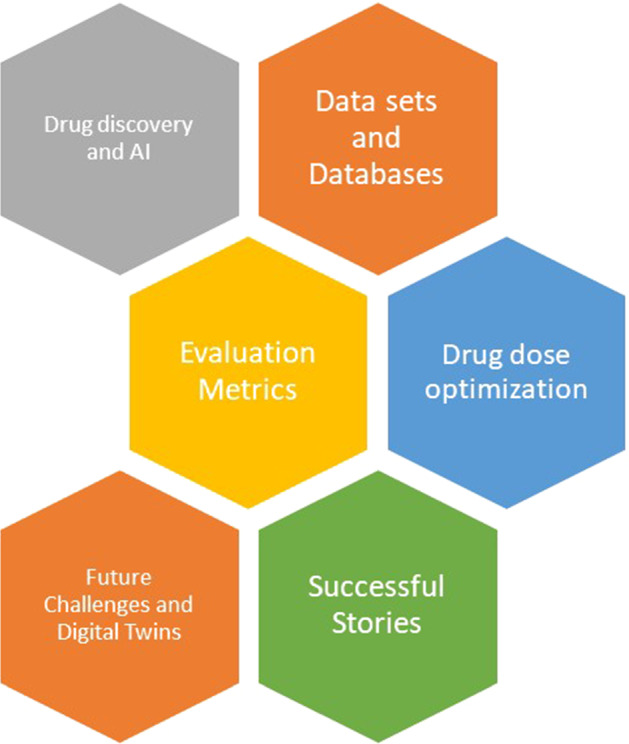
The main building blocks of the paper

The paper is developed based on six building blocks as shown in Fig. 1. More than 300 articles are presented in this paper, and they are divided across these building blocks. The papers are selected using the following criteria:

- The papers which published from 2000 to 2022.
- The papers which published in IEEE, ACM, Elsevier, and Springer have more priority.

The following analytical questions are discussed and completely being answered in the paper:

- AQ1: What DL algorithms have been used to predict the different categories of drug discovery problems?
- AQ2: Which deep learning methods are mostly used in drug dosing optimization?
- AQ3: Are there any success stories about drug discovery and DL?
- AQ4: What about the newest technologies such as XAI and DT in drug discovery?
- AQ5: What are the future and open works related to drug discovery and DL?

The remainder of this review paper is organized as: Sect. 2 presents a review of related studies; Sect. 3 covers the various DL techniques as an overview. Section 4 presents the organization of DL applications in drug discovery problems through explaining each drug discovery problem category and gives a literature review of the DL techniques used. Section 5 discusses the numerous benchmark data sets and databases that have been employed in the drug development process. Section 6 presents the evaluation metrics used for each drug discovery problem category. The drug dose optimization, successful stories, and XAI are introduced in Sect. 7, Sect. 8, and Sect. 9. DT and open problems are suggested as future research challenges in Sects. 10 and 11. Section 12 presents a discussion of the analytical questions. Finally, Sect. 13 concludes the paper.

## Review of related studies

Although the drug discovery is a large field and has different research categories, there is a few review studies about this field and each related study has focused only on a one research category such as reviewing the DL applications for the DTIs. This section aims to review these related studies and a summary is presented in Table 1.

**Table 1:** Related studies included DL for drug discovery

Reference	Main context	Year	Category
Kim et al. (2021)	Survey of DL models in drug–target interaction (DTI) and new medication development	2021	DTI
Rifaioglu et al. (2019)	A discussion about the most recent uses of ML techniques in VS (DTI application), including DL	2019	DTI
Sachdev et al. (2019)	Survey of the feature based chemogenomic methods for DTIs prediction	2019	DTI

Kim et al. (2021) presented a survey of DL models in the prediction of drug–target interaction (DTI) and new medication development. They start by providing a thorough summary of many depictions of drugs and proteins, DL applications, and widely used exemplary data sets to test and train models. One good point for this study, they identify a few obstacles to the bright future of de novo drug creation and DL-based DTI prediction. However, the major drawback of this study was that it did not consider the latest technology in DL application for the DTIs such as XAI and DTs.

Rifaioglu et al. (2019) presented the recent ML applications in Virtual Screening (VS) with the techniques, instruments, databases, and materials utilized to create the model. They outline what VS is and how crucial it is to the process of finding new drugs. Good points for this study, they highlighted the DL technologies that are accessible as open access programming libraries and provided instances of VS investigations that resulted in the discovery of novel bioactive chemicals and medications, tool kits and frameworks, and can be employed for the foreseeable future's computational drug discovery (including DTI prediction). However, they did not consider the drug dose optimization in their literature review.

Sachdev and Gupta (2019) presented the various feature based chemogenomic methods for DTIs prediction. They offer a thorough review of the different methodologies, datasets, tools, and measurements. They give a current overview of the various feature-based methodologies. Additionally, it describes relevant datasets, methods for determining medication or target properties, and evaluation measures. Although the study considered the initial integrated review which concentrate only on DTI feature-based techniques, they did not consider the latest technology in DL application for the DTIs such as XAI and DTs.

## Deep learning (DL) techniques

Detecting spam, recommending videos, classifying images, and retrieving multimedia ideas are just a few of the techniques used are just a few of the applications where machine learning (ML) has lately gained favor in research. Deep learning (DL) is one of the most extensively utilized ML methods in these applications. The ongoing appearance of new DL studies is due to the unpredictability of data acquisition and the incredible progress made in hardware technologies. DL is based on conventional neural networks but outperforms them significantly. Furthermore, DL uses transformations and graph technology to build multi-layer learning models (Kim et al. 2021). With their groundbreaking invention, Machine Learning and Deep Learning have revolutionized the world's perspective. Deep learning approaches have revolutionized the way we tackle problems. Deep learning models come in various shapes and sizes, capable of effectively resolving problems that are too complex for standard approaches to tackle. We'll review the various deep learning models in this section (Sarker 2021).

### Classic neural networks

**Fig. 2:**
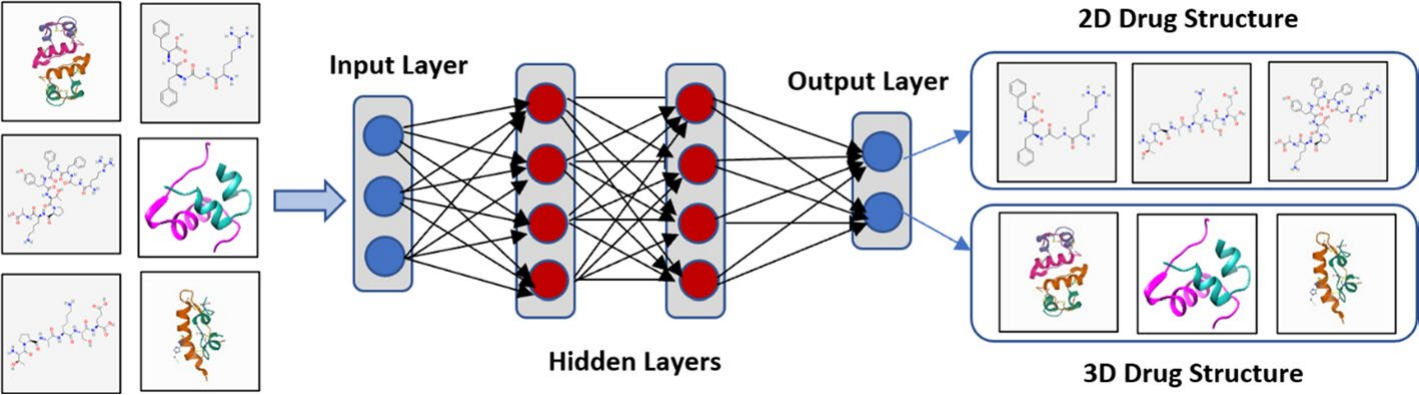
Multilayer Perceptron or ANN

As shown in Fig. 2, Multi-layer perceptron are frequently employed to recognize Fully Connected Neural Networks. It involves converting the algorithm into simple two-digit data inputs (Mukhamediev et al. 2021). This paradigm allows for both linear and nonlinear functions to be included. The linear function is a single line with a constant multiplier that multiplies its inputs. Sigmoid Curve, Hyperbolic Tangent, and Rectified Linear Unit are three representations for nonlinear functions. This model is best for categorization and regression issues with real-valued data and a flexible model of any kind.

### Convolutional neural networks (CNN)

**Fig. 3:**
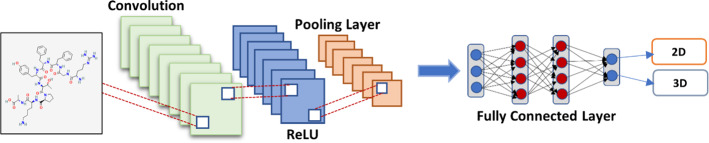
Convolutional Neural Networks (CNN)

As shown in Fig. 3, The classic convolutional neural network (CNN) model is an advanced and high-potential variant ANN Which developed to manage escalating complexity levels, as well as data pretreatment and compilation. It is based on how an animal's visual cortex's neurons are arranged (Amashita et al. 2018). One of the most flexible algorithms for the processing of data with and without images is CNNs. CNN can be processed through 4 phases:

- For analyzing basic visual data, such as picture pixels, it includes one input layer that is often the case a 2D array of neurons.
- Some CNNs analyze images on their inputs using a single-dimensional output layer of neurons coupled to distributed convolutional layers.
- Layer number 3, called as the sampling layer, is included in CNNs o restrict the number of neurons which It took part in the relevant network levels.
- The sampling and output layers are joined by one or more connected layers in CNNs.

This network concept can potentially aid in extracting relevant visual data in pieces or smaller units. In the CNN, the neurons are responsible for the group of neurons from the preceding layer.

After the input data has been included into the convolutional model, the CNN is constructed in four steps:

- Convolution: The method produces feature maps based on supplied data., which are then subjected to a purpose.
- Max-Pooling: It aids CNN in detecting an image based on supplied changes.
- Flattening: The data is flattened in this stage so that a CNN can analyze it.
- Full Connection: It's sometimes referred to as a "hidden layer" which creates the loss function for a model.

Image recognition, image analysis, image segmentation, video analysis, and natural language processing (NLP) (Chauhan et al. 2018; Tajbakhsh et al. May 2016; Mohamed et al. 2020; Zhang et al. 2018) are among the tasks that CNNs are capable of.

### Recurrent neural networks (RNNs)

**Fig. 4:**
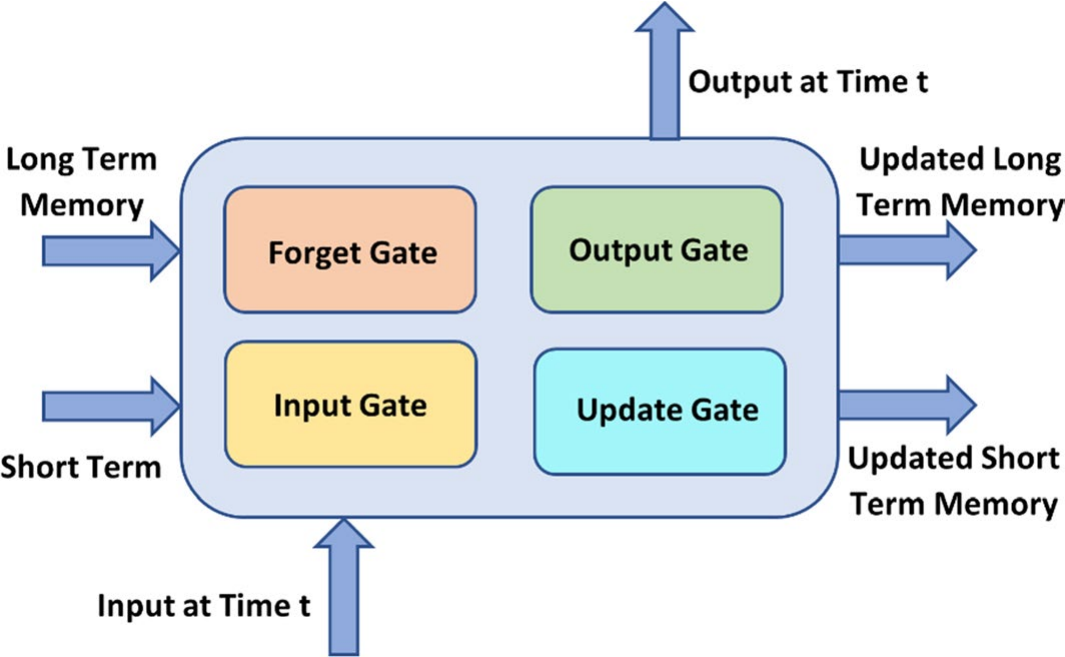
LSTM Network

RNNs were first created to help in sequence prediction. These networks rely solely on data streams with different lengths as inputs. For the most recent forecast, the knowledge of its previous state is used as an input value by the RNN. As a result, it can help a network's short-term memory achievers (Tehseen et al. 2019). As shown in Fig. 4, The Long Short-Term Memory (LSTM) method, for example, is renowned for its adaptability.

LSTMs, which are advantageous in predicting data in time sequences using memory, and LSTMs, which are useful in predicting data in time sequences using memory, are two forms of RNN designs that aid in the study of problems. The three gates are Input, Output, and Forget. Gated RNNs are particularly helpful for temporal sequence prediction using memory-based data. Both types of algorithms can be used to address a range of issues, including image classification (Chandra and Sharma 2017), sentiment analysis (Failed 2018), video classification (Abramovich et al. 2018), language translation (Hermanto et al. 2015), and more.

### Generative adversarial networks: GAN

**Fig. 5:**
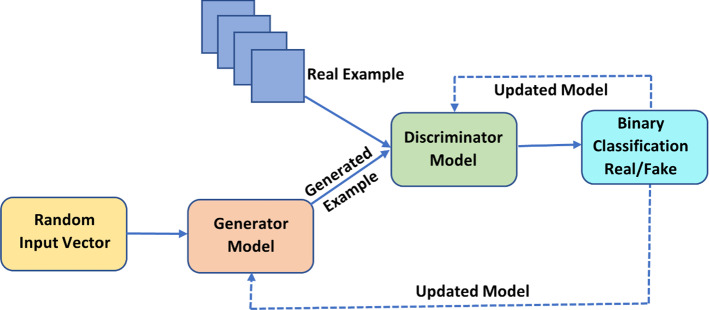
GAN: *Generative Adversarial Networks*

As shown in Fig. 5, It combines a Generator and a Discriminator DL neural network approach. The Discriminator helps to discriminate between real and fake data while the Generator Network creates bogus data (Alankrita et al. 2021).

Both networks compete with one another as The Discriminator still distinguishes between actual and fake data, and the Generator keeps making fake data look like real data. The Generator network will generate simulated data for the authentic photos if a picture library is necessary. Then, a deconvolution neural network would be created. Then, an Image Detector network would be utilized to discriminate between fictitious and real images. This competition would eventually help the network's performance. It can be employed in creating images and texts, enhancing the image and discovering new drugs.

### Self-organizing maps (SOM)

**Fig. 6:**
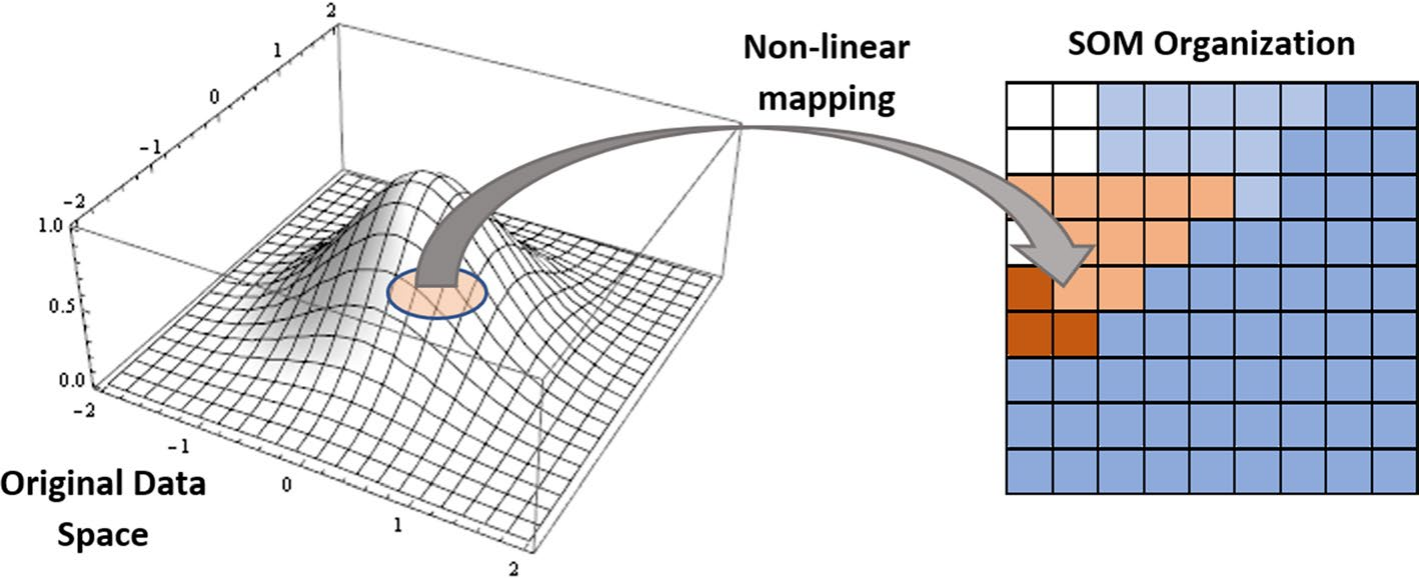
Self-Organizing Maps (SOM)

As shown in Fig. 6, Self-Organizing Maps operate by leveraging unsupervised data to decrease a model's number of random variables (Kohonen 1990). Given that every synapse is linked to both its input and output nodes, the output dimension in this DL approach is set as a two-dimensional model. The competition between each data point and its model representation in the Self-Organizing Maps, the weight of the closest nodes or Best Matching Units is adjusted (BMUs). The value of the weights varies based on how close a BMU is. The value represents the node's position in the network because weights are a node attribute in and of themselves. It's great for evaluating dataset frameworks that don't have a Y-axis value or project explorations that don't have a Y-axis value.

### Boltzmann machines

**Fig. 7:**
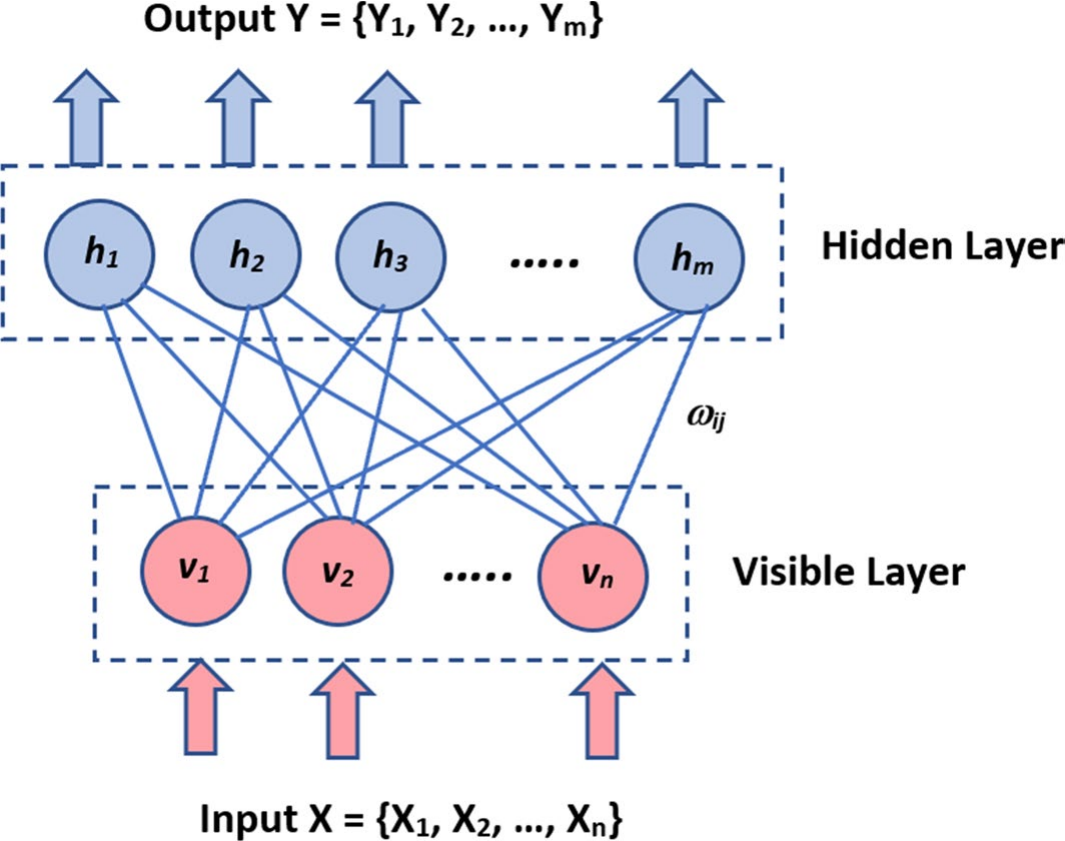
Boltzmann Machines

As shown in Fig. 7, the nodes are connected in a circular pattern because there is no set orientation in this network model. This deep learning technique is utilized to generate model parameters because of its uniqueness. The Boltzmann Machines model is stochastic, unlike all preceding deterministic network models. It can monitor systems, create a binary recommendation platform, and analyze specific datasets (Hinton 2011).

The architecture of the Boltzmann Machine is a two-layer neural network. The visible or input layer is the first, while the hidden layer is the second. They are made up of several neuron-like nodes that carry out computations. These nodes are interconnected at different levels but are not linked across nodes in the same layer. As a result, there is no connectivity between layers, which is one of the Boltzmann machine's disadvantages. When data is supplied into these nodes, it is transformed into a graph, and they process it and learn all the parameters, motifs, and relations between them before deciding whether to transmit it. As a result, an Unsupervised DL model is often known as a Boltzmann Machine.

### Autoencoders

**Fig. 8:**
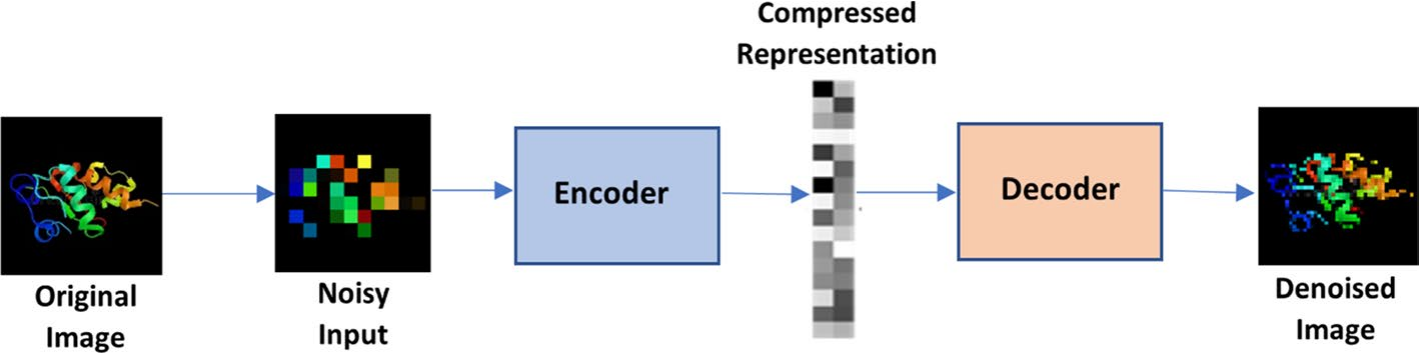
Autoencoders

As shown in Fig. 8, This algorithm, one of the most popular deep learning algorithms, automatically based on its inputs, applies an activation function, and decodes the result at the end. Because of the backlog, there are fewer types of data produced, and the built-in data structures are used to their fullest extent (Zhai et al. 2018).

There are various types of autoencoders:

- Sparse: The generalization technique is used when the hidden layers outnumber the input layer to decrease the overfitting. It constrains the loss function and restricts the autoencoder from utilizing all its nodes simultaneously.
- Denoising: In this case, randomly, the inputs are adjusted and made to equal 0.
- Contractive: When the hidden layer outnumbers the input layer, to avoid overfitting and data duplication, a penalty factor is introduced to the loss function.
- Stacked: When another hidden layer is added to an autoencoder, it results in two stages of encoding and Initial stages of decoding.

Feature identification, establishing a strong recommendation model, and adding features to enormous datasets are some of the difficulties it can solve.

## Organization of DL applications in drug discovery problems

**Fig. 9:**
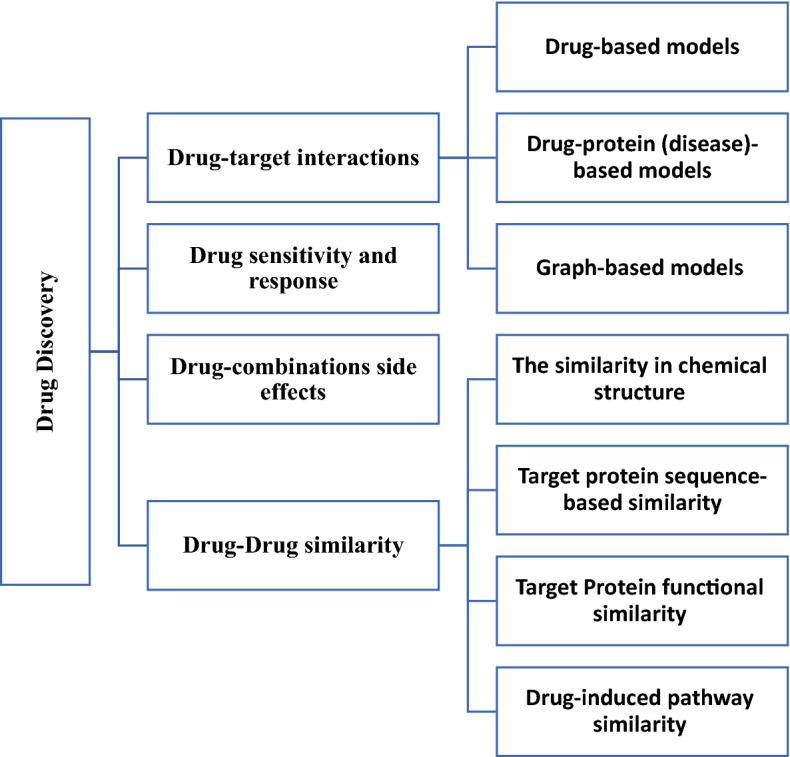
Drug discovery problem categories

The evolution of safe and effective treatments for human is the primary goal of drug discovery (Kim et al. 2021). Drug discovery is the problem of finding the suitable drugs to treat a disease (i.e., a target protein) which relies on several interactions. This paper divides the drug discovery problems into four main categories, as presented in Fig. 9. They are drug–target interactions, drug–drug similarity, drug combinations side effects, and drug sensitivity and response predictions. The following subsections provide a literature review of DL with these problems and some of the investigated literature articles related to each category are summarized in Table 2.

**Table 2:** Classification of articles related to drug discovery and DL

References	Publishing year	Method	Advantages	Drawbacks
Drug–target interactions (DTIs)				
Yang et al. (2019)	2019	A graph convolutional model that consistently equals or outperforms models with constant molecular descriptors and earlier graph neural designs on both open-source and closed-source data sets	The model performs equally well or better on 12 of the 19 open data sets	The proposed model is underperforming other models when either (1) the 3D information from the other models, (2) the data set is especially small, or (3) the classes are particularly imbalanced
Hirohara et al. (2018)	2020	A brand-new CNN for assessing data about chemical compounds. The CNN processes picture data similarly to traditional CNNs using a SMILES-based feature matrix	The standard fingerprint method used for the virtual screening of chemical compounds performed worse than the CNN based on SMILES string. Additionally, the use of motif detection with learnt filters allowed the detection of undiscovered functional group substructures in addition to significant recognised substructures like protein-binding sites	The model performed better than DNN using simply ECFP in the TOX 21 Challenge 2014 experiment, but somewhat worse than models utilising both ECFP and DeepTox features
Shin et al. (2019)	2019	A novel representation of a molecule employing the self-attention method that has been pre-trained using massive data on chemicals that is available to the public	Regarding four evaluation metrics, the model performs better than all other techniques now in use. Furthermore, the strategy is successful in including all the existing EGFR medications in the top-30 promising prospects, as demonstrated by the example study of discovering drug candidates targeting a cancer protein (EGFR)	The protein was ignored by the model. One explanation is because, on average, a protein sequence is 10 times longer than a molecule sequence, which requires a significant amount of computation time. Another justification is the requirement of a protein dataset with adequate data to pre-train the model
Lee et al. (2019)	2019	A new DTI prediction model by identifying regional residue trends from the complete sequences of the target protein via CNN	Other protein descriptors, such as CTD and SW scores, do not perform as well as the local properties of protein sequences that have been found. Compared to techniques requiring 3D structures, it can be more broadly utilized to predict DTIs	To attain enhanced performance, more detailed chemical characteristics are used for DTI forecasting. Chemical elaboration can be replaced by the consideration of 3D structure information
Wang et al. (2020b)	2020	A novel method MDADTI to predict DTIs based on MDA	MDA lowered the size of the characteristic of medications and objectives, which accelerate the training of MDADTI	Did not consider predicting the binding affinity scores for drug–target pairs
Wan et al. (2019)	2019	DeepCPI is a new and scalable approach that predicts innovative CPIs by combining data-driven representation learning and DL (DTIs)	To effectively utilize the enormous amount of data on the interactions between compounds and proteins accessible from expansive databases, including PubChem and ChEMBL, the synthesis of the efficient feature embedding methodologies with the potent DL model is especially helpful	Better prediction results may be reached by combining other accessible data, like as gene expression and protein structures, into the suggested DL model
Vazquez et al. (2020)	2020	Model for defining hybrid LB (ligand-based) + SB (structure-based) computational schemes in VS (Virtual screening) studies	VS has been a powerful alternative to high-throughput screening assays. The performance of VS campaigns is significantly impacted by the inherent weaknesses of both LB and SB procedures while being the most popular way for exposing innovative hit compounds in the early phases of drug discovery	Although the progress is encouraging, it can be assumed that two primary elements will determine if these integrated tactics are adopted. Initially, a thorough benchmarking of the various combination tactics Secondly, convenient access to screening of focused chemical library searches should be made possible by the capacity to integrate the combined LB and SB techniques into automated modelling platforms
Xie et al. (2018)	2018	A framework that offers better prospects for inferencing and for DTI prediction based on L1000 database transcriptome data from pharmacological perturbation and gene knockdown experiments	The outcomes showed that our system can find DTIs that are more trustworthy than those discovered by previous techniques	investigating a possibility to discover an internal characteristic for assessing fresh DTI potential
Drug sensitivity and response				
Dincer et al. (2018)	2018	A low-dimensional representation (LDR) is encoded using the DeepProfile framework, which learns a variational autoencoder (VAE) network using tens of thousands of publicly available gene expression samples. This network is then used to predict complicated disease symptoms	DeepProfile successfully disentangles data inconsistencies and discovers a useful LDR that precisely foretells complicated phenotypes of many malignancies	The work needs some future directions which include: (1) training DeepProfile using samples from various cancers and (2) expanding the application of DeepProfile by utilizing multi-omics data to produce more insightful embeddings for cancer
Rampášek et al. (2019)	2019	‘Dr.VAE’, the first unified machine learning method for the semi-supervised learning for drug response prediction that successfully exploits the prior knowledge of drug-induced transcriptomic perturbations	First, the flexibility of This paradigm made it possible to incorporate the effects of transcriptional disruption into the framework for predicting medication reaction in a special way Second, neural networks with the ability to simulate complex non-linear interactions are employed to parametrize all conditional distributions that make up their model, in addition to variational posteriors employed by Dr. VAE to make a rough inference	The study could be limited in numerous ways. As it has repeatedly been demonstrated to offer the greatest predictive potential in several prior research on treatment response, they first just took the gene expression modality into consideration. Second, they modeled CMap-L1000v1 perturbations following a 6-h treatment period. Since many feedback regulatory processes take 6 h to appear, it may be argued that these studies alone do not give a complete picture of the transcriptome response
Ahmed et al. (2020)	2020	Two neural network models based on graphs for predicting drug sensitivity as well as a network-based feature selection approach	To predict drug sensitivity, using a network to pick features. first identifies additional illustrative characteristics based on the network of gene co-expression. Second, Random Forest performs better than all the other established prediction techniques., Third, compared to DNN, the graph-based neural network models exhibit higher drug response prediction capability., Fourth, the performance of the prediction is reliant on the drug and could be related to the drug's mode of action (MOA)	In this study, there was little improvement for DNNs using graphs. Multi-omics data can be used for analysis to further improve drug prediction accuracy. In addition to the extensive biological features, data acquisition using multi-omics the genomic, epigenomic, and transcriptomic traits of each cell line in the cohort and offer more precise molecular fingerprints to forecast medication reaction than single-omics data alone
Ren et al. (2022)	2022	DeepGRMF, which demonstrated enhanced capability for predicting drug sensitivities	model can predict new responses to drugs or medications not previously seen, which It makes it possible to use already FDA approved medications in new ways to both cure cancer and maybe find new molecules that fight cancer	the DeepGRMF model only uses data from gene expression profiling, incorporating genetic changes further and epigenetic data will probably help the model perform even better
Drug–drug interactions (DDIs) side effect				
Jae et al. (2018)	2018	DeepDDI that accurately forecasts DDI types for medication combinations as well as drug-food component pairings using as inputs names and chemical structures	According to the seven common performance criteria, DeepDDI's feature vector of pharmaceuticals, which combines an improved DNN and SSP, demonstrated great accuracy (84.8–93.2%)	Only two medications are included in the best DDI database provided by DrugBank. The DNN can be updated by training DeepDDI for numerous medications and food ingredients once DDI data for multiple drugs and/or food ingredients becoming accessible
Xiang et al. (2020)	2020	An overview of various graph embedding approaches is provided, along with an assessment of how well they perform on link prediction and node categorization, two significant biological tasks	Through comprehensive testing, they discover that current graph embedding techniques can compete favorably with state-of-the-art techniques or even outperform them in a variety of biological prediction tasks	The idea of pre-training followed by fine-tuning becomes increasingly intriguing as there are more entities that may have pre-trained embeddings as biomedical data volume increases. The relationships between the freshly created network propagation (diffusion) methods and the graph embedding techniques may also have an impact on future research
Xifan et al. (2020)	2020	A multimodal deep learning system called DDIMDL uses deep learning and a variety of pharmacological characteristics to forecast DDI events	The experimental findings demonstrate that DDIMDL performs better than the approaches that were examined in terms of efficiency and accuracy	to enhance the forecast of DDI events. First, there needs to be consideration of unique strategies for dealing with the unbalanced dataset due to the extreme imbalance in the amount of DDIs for various occurrences. Second, certain events have insufficient numbers of interactions, making it easier for the DL approach to underfit, hence techniques to increase the size of the event dataset can be investigated
Karim et al. (2019)	2019	A new ML approach for forecasting DDIs using a variety of sources	The incorporation of drug data from many sources using knowledge graphs. This candidate will get comprehensive background information on medications, illnesses, processes, proteins, enzymes, chemical structures, etc	The approach's inability to offer reasons for the projected DDIs is one of its limitations
Bongini et al. (2022)	2022	Graph Neural Networks (GNNs) are exploited to predict DSEs	A GNN-based predictor aids in foreseeing the incidence of adverse effects. Additionally, its use with novel candidate medications would assist drug discovery studies save time and money while avoiding health problems for the volunteers taking part in clinical trials	An exciting future path is the creation of a prediction based on a GNN that It has the capacity to analyze molecular structural formulae, which are displayed as graphs. Features could be added to these molecular graphs to improve them by way of genes and links between genes and drugs
Iorio et al. (2010)	2010	A broad method to identify previously unknown uses for well-known medications as well as to forecast the molecular actions and MOA of new compounds	Using information buried in the gene expression profiles after drug treatment to identify pharmacological MoA similarities	The approach's primary drawback is the network's small number of chemicals. If a chemical is not like any of the medications in the network, no inferences about its MoA or its biological effects can be made because the approach is predicated on comparing how similar two drugs are
Wang et al. (2019)	2019	An approach using networks to find clinically effective drug combinations for disorders	Provides a network-level analysis of the comparative efficacy and harmful interactions of treatment combinations	The applicability of their findings to different disorders must be investigated in further research
Heba et al. (2021)	2021	The ML-based on similarity for drug interaction prediction (SMDIP) framework, which incorporates popular ML models utilizing internal similarity models based on products to choose the characteristics and benefit from more sparse feature space to enhance DDI prediction efficiency	creating a generalizable machine learning framework based on similarities to reveal innovative DDIs having excellent forecasting abilities; and (2) Showcasing superior predictor elements in comparison to the body of existing research	SMDIP exhibits some restrictions. SMDIP is first and foremost a binary classifier framework with DDI results. It cannot, however, predict how serious a DDI will be. Second, SMDIP cannot make recommendations for the best course of action to be done for the management of DDI since it cannot forecast the unfavorable effects brought on by the interaction

### Drug–target interactions prediction using DL

Drug repurposing attempts to uncover new uses for drugs that are already on the market and have been approved. It has attracted much attention since it takes less time, costs less money, and has a greater success rate than traditional de novo drug development (Thafar et al. 2022). The discovery of drug–target interactions is the initial step in creating new medications, as well as one of the most crucial aspects of drug screening and drug-guided synthesis (Wang et al. 2020a). Exploring the link between possible medications and targets can aid researchers in better understanding the pathophysiology of targets at the drug level, which can help with the disease's early detection, treatment prognosis, and drug design. This is well known as drug–target interactions (DTIs) (Lian et al. 2021). Achieving success to the drug repositioning mechanism largely reliant on DTI's forecast because it reduces the number of potential medication candidates for specific targets. The approaches based on molecular docking and the approaches based on drugs are the two basic tactics used in traditional computational methods. When target proteins' 3D structures aren't available, the effectiveness of molecular docking is limited. When there are only a few known binding molecules for a target, drug-based techniques typically produce subpar prediction results. DL technologies overcome the restrictions of the high-dimensional structure of drug and target protein by using unstructured-based approaches which do not need 3D structural data or docking for DTI prediction. Therefore, this section provides a recent comprehensive review of DL-based DTIs prediction models (Chen et al. 2012).

**Fig. 10:**
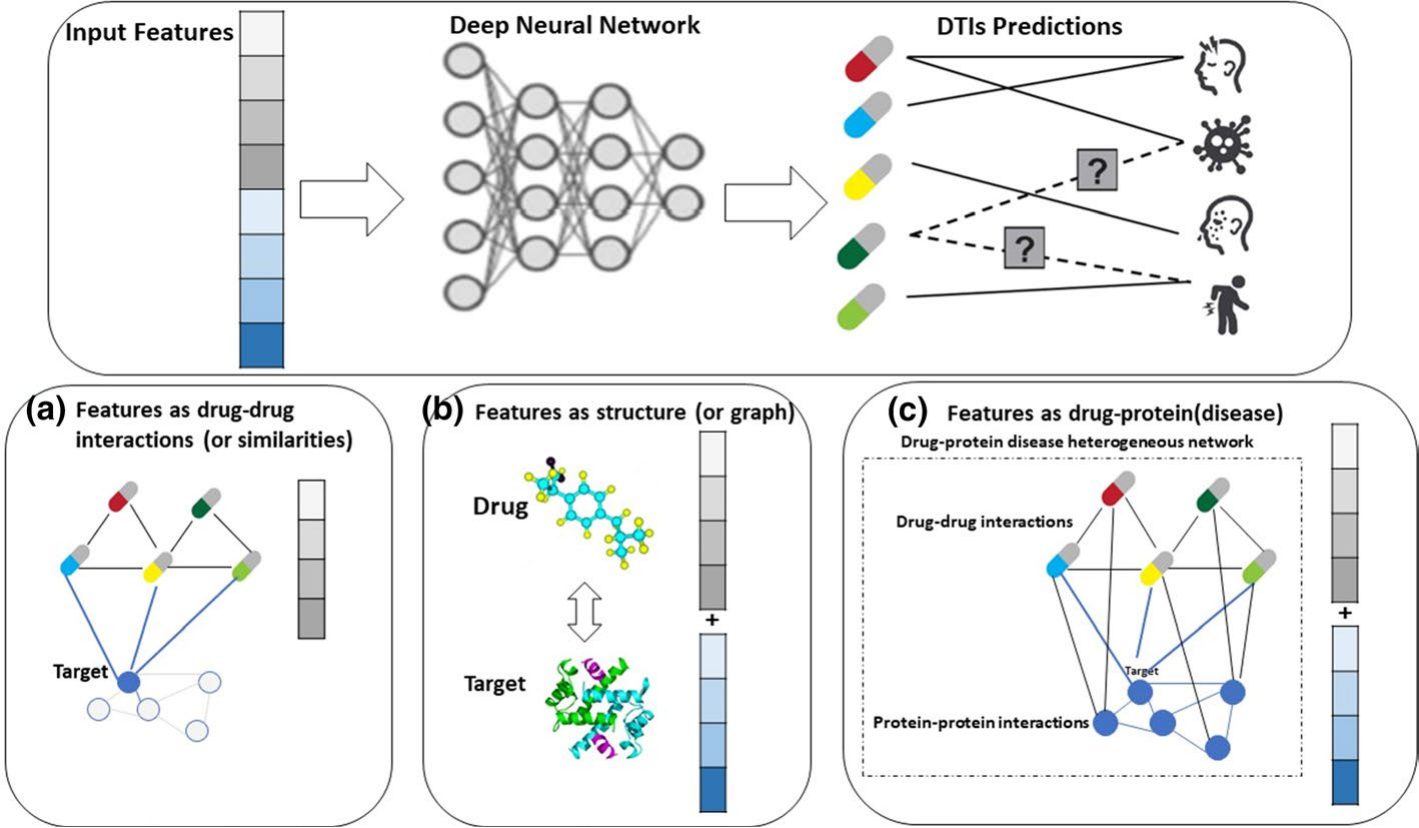
DL models used for predicting the DTIs are grouped into three categories: **a** drug-based models, **b** structure (graph)-based models, and **c** drug-protein(disease)-based models

As shown in Fig. 10, there are known interactions (solid lines) and unknown interactions (dashed lines) between diseases (proteins) and drugs. DTIs forecast unknown interactions or what diseases (or target proteins) a new drug might treat. According to their input features, we divided the latest DL models used to predict DTIs into three categories: drug-based models, structure (graph)-based models, and drug-protein(disease)-based models.

#### Drug-based models

Figure 10A shows drug-based models that assume a potential drug will be like known drugs for the target proteins. It calculates the DTI using the target's medication information. Similarity search strategies are used in these models, which postulate that structurally similar substances have similar biological functions (Thafar et al. 2019; Matsuzaka and Uesawa 2019). These methods have been used for decades to select compounds in vast compound libraries employing massive computer jobs or solve problems using human calculations. Deep neural network models gradually narrow the gap between in silico prediction and empirical study, and DL technology can shorten these time-consuming procedures and manual operations.

Researchers may now use deep neural networks to analyze medicines and predict drug-related features, including as bioactivities and physicochemical qualities, thanks to using benchmark packages like MoleculeNet (Wu et al. 2018) and DeepChem (). As a result, basic neural networks like MLP and CNN have been used in numerous drug-based DL approaches (Zeng et al. 2020; Yang et al. 2019; Liu et al. 2017). The representation power of molecular descriptors was often the focus of ADMET investigations rather than the model itself (Zhai et al. 2018; Liu et al. 2017; Kim et al. 2016; Tang et al. 2014). Hirohara et al. trained a CNN model with the SMILES string and then used learned attributes to discover motifs using significant structures for locations that bind proteins or unidentified functional groupings (Hirohara et al. 2018). Atom pairs and pharmacophoric donor–acceptor pairings have been employed by Wenzel et al. (2019) as adjectives in multi-task deep neural networks to predict microsomal metabolic liability. Gao et al. (2019) compared 6 different kinds of 2D fingerprints in the prediction of affinity between proteins and drugs using ML methods such as RF, single-task DNN, and multi-task DNN models. Matsuzaka and Uesawa (2019) used 2D pictures of 3D chemical compounds to train a CNN model to predict constitutive androstane receptor agonists. They optimized the greatest performance in snapshots of a 3D ball-and-stick model taken at various angles or coordinates. Therefore, the method outperformed seven common 3D chemical structure forecasts.

Since the GCN's development, drug related GCN models have created depictions of graphs which concerned with molecules that incorporate details on the chemical structures by adding up the adjacent atoms' properties (Gilmer et al. 2017).

GCNs have been employed as 3D descriptors instead of SMILES strings in a lot of research, and it's been discovered that these learned descriptors outperform standard descriptors in prediction tests and are easier to understand (Shin et al. 2019; Ozturk et al. 2018; Yu et al. 2019). Chemi-net employed GCN models to represent molecules and compared the performance of single-task and multi-task DNNs on their own QSAR datasets (Liu et al. 2019a). Yang et al. (2019) introduced the directed message passing neural network, which uses a directed message-passing paradigm, as a more advanced model (D-MPNN). They tested their approaches on 19 publicly available and 16 privately held datasets and discovered that in most situations, they were correct. The D-MPNN models outperformed the previous models. In two datasets, they underperformed and were not as resilient as typical 3D descriptors when the sample was small or unbalanced. The D-MPNN model was then employed by another research group to correctly forecast a kind of antibiotic named HALICIN, which demonstrated bactericide effects in models for mice (Stokes et al. 2020). This was the first incident that resulted in the finding of an antibiotic by using DL methods to explore a large-scale chemical space that current experimental methodologies cannot afford. The application of attention-based graph neural networks is another interesting contemporary method (Sun et al. 2020a). Edge weights and node features can be learned together since a molecule's graph representations can be altered by edge properties. As a result, Shang et al. suggested a multi-relational GCN with edge attention (Shang et al. 2018). For each edge, they created a reference guide on attention spans. Because it is used throughout the molecule, the approach can handle a wide range of input sizes.

In the Tox21 and HIV benchmark datasets, they found that this model performed better than the random forest model. As a result, the model may effectively learn pre-aligned features from the molecular graph's inherent qualities. Withnall et al. (2020) extended the MPNN model with AMPNN (attention MPNN), which is an attention technique that the message forwarding step employs weighted summation. Moreover, they termed the D-MPNN model the edge memory neural network because it was extended by the same attention mechanism as the AMPNN (EMNN). Although it is computationally more intensive than other models, this model fared better than others on the uniformly absent information from the maximal unbiased validation (MUV) reference.

#### Structure (graph)-based models

Unlike the drug- and structure-based models in Fig. 10b, protein targets and medication information should be included. Typical molecular docking simulation methods aim to predict the geometrically possible binding of known tertiary structure drugs and proteins. Atom sequences and amino acid residues can be used to express both the medicine as well as the target. Descriptors based on sequences were selected because DL approaches may be implemented right away with non-significant pre-processing of the entering data.

The Davis kinase binding affinity dataset (Davis et al. 2011) and the KIBA dataset (Sun et al. 2020a) were used in that study. DeepDTA, suggested by Ozturk et al. (2018), outperformed moderate ML approaches such as KronRLS (Nascimento et al. 2016) and SimBoosts (Tong et al. 2017) by applying solely information about the sequence of a CNN model based on the SMILES string and amino acid sequences. Wen et al. used ECFPs and protein sequence composition descriptors as examples of common and basic features and trained them using semi-supervised learning via a deep belief network (Wen et al. 2017). Another study, DeepConv-DTI, built a deep CNN model using only an RDKit Morgan fingerprint and protein sequences (Lee et al. 2019). They also used the pooled convolution findings to capture local residue patterns of target protein sequences, resulting in high values for critical protein areas like actual binding sites.

The scoring feature, which ranks the protein-drug interaction with 3D structures and makes the training data parametric to forecast values for binding affinities of targeted proteins, is used to predict binding affinity values or binding pocket sites of the target proteins as a key metric for the structure-based regression model. The protein–drug complexes' 3D structural characteristics were included in the CNNs by AtomNet (Wallach et al. 2015). They placed 3D grids with set sizes (i.e., voxels) in comparison to protein–drug combinations, with every cell in the grid representing structural properties at that position. Several researchers have examined the situation since then, deep CNN models that use voxels to predict binding pocket location or binding affinity (Wang et al. 2020b; Ashburner et al. 2000; Zhao et al. 2019). In comparison to common docking approaches such as AutoDock Vina (Trott and Olson 2010) or Smina (Koes et al. 2013), these models have shown enhanced performance. This is since CNN models are relatively impervious even with large input sizes. It can be taught and is resilient to input data noise.

Many DTI investigations using GCNs based on structure-based approaches have been reported (Feng et al. 2018; Liu et al. 2016). Feng et al. (2018) used both ECFPs and GCNs as pharmacological characteristics. In the Davis et al. (2011), Metz et al. (2011), and KIBA Tang et al. (2014) benchmark datasets, their methods outperformed prior models such as KronRLS (Nascimento et al. 2016) and SimBoost (Tong et al. 2017). However, they did agree that their GCN model couldn't beat their ECFP model due to time and resource constraints in implementing the GCN. In a different DTI investigation study, Torng et al. employed a graph without supervision to become familiar with constant size depictions of protein binding sites (Torng and Altman 2019). The pre-trained GCN model was then trained using the newly created protein pocket GCN, the drug GCN model, on the other hand, used attributes to be trained and which were generated automatically. They concluded that without relying on target–drug complexes, their model effectively captured protein–drug binding interactions.

Because the models that implement the attention mechanism have key qualities that enable the model to be interpreted, attention-based DTI prediction approaches have evolved (Hirohara et al. 2018; Liu et al. 2016; Perozzi et al. 2014).

For protein sequences, Gao et al. (2017) employed compressed vectors with the LSTM RNNs and the GCN for drug structures. They concentrated on demonstrating their method's capacity to deliver biological insights into DTI predictions. To do so, Mechanisms for two-way attention were employed. to calculate the binding of drug–target pairs (DTPs), allowing for flexible interpretation of superior data from target proteins, such as GO keywords. Shin et al. (2019) introduced the Molecule transformer DTI (MT-DTI) approach for drug representations, which uses the self-attention mechanism. The MT-DTI model was tweaked to perfection and assessed using two Davis models Using pre-trained parameters from the 97 million chemicals PubChem (Davis et al. 2011) and (KIBA) (Tang et al. 2014) benchmark datasets, which are both publicly available. However, the attention mechanism was not used to depict the protein targets because it would take too long to calculate the target sequence in an acceptable amount of time. Pre-training is impossible due to a lack of target information.

On the other hand, attention DTA presented by Zhao et al. incorporates a CNN attention mechanism model to establish the weighted connections between drug and protein sequences (Zhao et al. 2019). They showed that these attention-based drug and protein representations have good MLP model affinity prediction task performance. DeepDTIs used external, experimental DTPs to infer the probability of interaction for any given DTP. Four of the top ten predicted DTIs have previously been identified, and one was discovered to have a poor glucocorticoid receptor binding affinity (Huang et al. 2018). DeepCPI was used to predict drug–target interactions. Small-molecule interactions with the glucagon-like peptide one receptor, the glucagon receptor, and the vasoactive intestinal peptide receptor have been tested in experiments (Wan et al. 2019).

#### Drug–protein(disease)-based models

According to poly pharmacology, most medicines have multiple effects on both primary and secondary targets. The biological networks involved, as well as the drug's dose, influence these effects. As a result, the drug–protein(disease)-based models shown in Fig. 10c are particularly beneficial when evaluating protein promiscuity or drug selectivity (Cortes-Ciriano et al. 2015). Furthermore, Neural networks that can do multiple tasks are ideal for simultaneously learning the properties of many sorts of data (Camacho et al. 2018). Several DL model applications, such as drug-induced gene-expression patterns and DTI-related heterogeneous networks, leverage relational information for distinct views. A network-based strategy employs heterogeneous networks includes a variety of nodes and edges kinds (Luo et al. 2017; David et al. 2019). The nodes in these networks have a local similarity, which is a significant aspect of these models. One can anticipate DTIs using their connections and topological features when a network of similarity with medications as its nodes and drug–drug similarity values as a measure of the edges' weights is investigated. Machine to support vectors (Bleakley and Yamanishi 2009; Keum and Nam 2017), Machine learning techniques that use heterogeneous networks as prediction frameworks include the regularized least square model (RLS) (Liu et al. 2016; Xia et al. 2010; Hao et al. 2016) and random walk with the restart model Nascimento (Lian et al. 2021; Nascimento et al. 2016). DTI prediction research using networks have employed DL to enhance the methods used to forecast associations today for evaluating the comparable topological structures of drug and target networks that are bipartite and tripartite linked networks, owing to the increased interest in the usage of DL technologies (drug, target, and disease networks) (Hassan-Harrirou et al. 2020; Lamb et al. 2006; Korkmaz 2020; Townshend et al. 2012; Vazquez et al. 2020). Zong et al. (2017) used the DeepWalk approach to collect local latent data, compute topology-based similarity in tripartite networks, and demonstrate the technology's promise as a medication repurposing solution.

Relationship-based features collected by training the AE were used in some network-based DTI prediction studies. Zhao et al. (2020) developed a DTI-CNN prediction model that combined depth information that is low-dimensional but rich with a heterogeneous network that has been taught using the stacked AE technique. To construct the topological similarity matrix of drug and target, Wang et al. used a deep AE and mutually beneficial pointwise information in their analysis (Wang et al. 2020b). Peng et al. (2020) employed a denoising Autoencoder to pick network-based attributes and decrease the representation dimensions in another investigation.

By helping the self-encoder learn to denoise, the anti-aliasing effect (Autoencoder) enhances high-dimensional images with noise, input data that is noisy and incomplete, allowing the encoder to learn more reliably. These approaches, however, have a drawback in that it is challenging to foresee recent medications or targets, a problem. The problem of recommendation systems' "cold start" is known as the "cold start" problem (Bedi et al. 2015). The size and form of the network have a big impact on these models, so if the network isn't big enough, they will not be able to collect all the medications or targets that aren't in the network (Lamb et al. 2006).

Various investigations have also utilized Gene expression patterns as chemogenomic traits to predict DTIs. This research presumes that medications with similar expression patterns have similar effects on the same targets (Hizukuri et al. 2015; Sawada et al. 2018).

The revised version of CMAP, the LINCS-L1000 database, has been integrated into the DL DTI models in recent works (Subramanian et al. 2017; Thafar et al. 2020; Karpov et al. 2020; Arus-Pous et al. 2020). Based on the LINCS pharmacological perturbation and knockout gene data, using a deep neural network, Xie et al. developed a binary classification model (Xie et al. 2018).

On the other hand, Lee and Kim employed as a source of expression signature genes medication and target features. They used node2vec to train the rich data by examining three elements of protein function, including pathway-level memberships and PPI (Lee and Kim 2019). Saho and Zhang employed a GCN model to extract drug and target attributes from LINCS data and a CNN model to forecast DTPs by extracting latent features in DTIGCCN (Shao et al. 2020). The Gaussian kernel function was identified to aid in the production of high-quality graphs, and as a result, this hybrid model scored better on classification tests.

DeepDTnet employs a heterogeneous drug–gene-disease network to uncover known drug targets containing fifteen types of chemicals and genomic, phenotypic, and cellular network properties. DeepDTnet predicted and experimentally confirmed topotecan, a new direct inhibitor of the orphan receptor linked to the human retinoic acid receptor (Zeng et al. 2020).

### Drug sensitivity and response prediction using DL

**Fig. 11:**
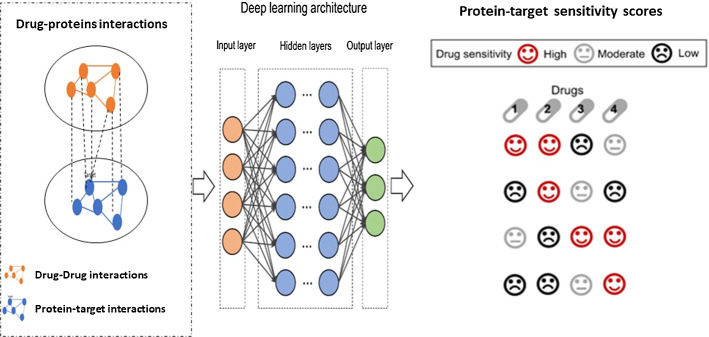
Drug binding with proteins and drug sensitivity (response) scores prediction

Drug response is the clinical outcome treated by the drug of interest (https://www.sciencedirect.com/topics/drug-response). This is due to the normally low ratio of samples to measurements each sample, which makes traditional feedforward neural networks unsuitable. The main idea of drug response prediction is shown in Fig. 11. The DL method takes the heterogenous network of drug and protein interactions as inputs and predicts the response scores. Although the widespread use of the deep neural network (DNN) approaches in various domains and sectors, including related topics like computational chemistry (Gómez-Bombarelli et al. 2018), DNNs have only lately made their way into drug response prediction. Overparameterization, overfitting, and poor generalization are common outcomes of recent simulation datasets. However, more public data has become available recently, and freshly built DNN models have shown promise. As a result, this section summarizes current DL computational problems and drug response prediction breakthroughs.

Since the 1990s, neural networks have been used to predict drug response (El-Deredy et al. 1997) revealed that data from tumor nuclear magnetic resonance (NMR) spectra might be used to train a neural network and can be utilized to predict drug response in gliomas and offer information on the metabolic pathways involved in drug response.

In 2018, The DRscan model was created by Chang et al. (2018), and it uses a CNN architecture that was trained on 1000 drug reaction studies per molecule. Compared to other traditional ML algorithms like RF and SVM, their model performed much better. CDRscan's ability to incorporate genomic data and molecular fingerprints is one of the reasons it outperformed these baseline models. Furthermore, its convolutional design has been demonstrated to be useful in various machine learning areas. A neural network called an autoencoder attempts to recreate the original data from the compressed form after compressing its input. As proven by Way and Greene (2018), this is very useful for feature extraction, which condensed a gene expression profile with 5000 dimensions with a maximum of 100 dimensions, some of which revealed to significant characteristics such as the patient's sexual orientation or melanoma status. Using variational autoencoders, Dincer et al. (2018) created DeepProfile, a technique for learning a depiction of gene expression in AML patients in eight dimensions that is then fitted to a Lasso linear model for treatment response prediction with superior results to that of no extracting features.

Ding et al. (2018) proposed a deep autoencoder model for representation learning of cancer cells from input data consisting of gene expression, CNV, and somatic mutations.

In 2019, MOLI (Multi-omics Late Integration) (Sharifi-Noghabi et al. 2019) was a deep learning model that incorporates multi-omics data and somatic mutations to characterize a cell line. Three separate subnetworks of MOLI learn representations for each type of omics data. A final network identifies a cell's response as responder or non-responder based on concatenated attributes. Those methods share two characteristics: integrating multiple input data (multi-omics) and binary classification of the drug response. Although combining several forms of omics data can improve the learning of cell line status, it may limit the method's applicability for testing on different cell lines or patients because the model requires extra data beyond gene expression.

Furthermore, a certain threshold of the IC50 values should be set before binary classification of the drug response, which may vary depending on the experimental condition, such as drug or tumor types. Twin CNN for drugs in SMILES format (TCNNS) (Liu et al. 2019b) takes a one-hot encoded representation of drugs and feature vectors of cell lines as the inputs for two encoding subnetworks of a One-Dimensional (1D) CNN. One-hot encodings of drugs in TCNNS are Simplified Molecular Input Line Entry System (SMILES) strings which describe a drug compound's chemical composition. Binary feature vectors of cell lines represent 735 mutation states or CNVs of a cell. KekuleScope (Cortés-Ciriano and Bender 2019) adopts transfer learning, using a pre-trained CNN on ImageNet data. The pre-trained CNN is trained with images of drug compounds represented as Kekulé structures to predict the drug response.

Yuan et al. (2019) offer GNNDR, a GNN-based technique with a high learning capacity and allows drug response prediction by combining protein–protein interactions (PPI) information with genomic characteristics. The value of including protein information has been empirically proven. The proposed method offers a viable avenue for the discovery of anti-cancer medicines. Semi-supervised variational autoencoders for the prediction of monotherapy response were examined by the Rampášek et al. (2019). In contrast to many conventional ML methodologies, together developed a model for predicting medication reaction that took advantage of expression of genes before and after therapy in cell lines and demonstrated enhanced evaluation on a variety of FDA-approved pharmaceuticals. Chiu et al. (2019) trained a deep drug response predictor after pre-training autoencoders using mutation data and expression features from the TCGA dataset. The use of pretraining distinguishes their strategy from others. Compared to using only the labeled data, the pretraining process permits un-labelled data from outside sources, like TCGA, as opposed to just gene expression profiles obtained from drug reaction tests, resulting in a significant increase in the number of samples available and improved performance.

Chiu et al. (2019) and Li et al. (2019) used a combination of auto-encoders and predicted drug reactions in cell lines with deep neural networks and malignancies that had been gnomically characterized. To anticipate cell lines reactions to drug combinations, in https://string-db.org/cgi/download.pl?sessionId=uKr0odAK9hPs used deep neural encoders to link genetic characteristics with drug profiles.

In 2020, Wei et al. (2020) anticipate drug risk levels (ADRs) based on adverse drug reactions. They use SMOTE and machine learning techniques in their studies. The proposed framework was used to investigate the mechanism of ADRs to estimate degrees of drug risk and to assist with and direct decision-making during the changeover from prescription to over-the-counter medications. They demonstrated that the best combination, PRR-SMOTE-RF, was built using the above architecture and that the macro-ROC curve had a strong classification prediction effect. They suggested that this framework could be used by several drug regulatory organizations, including the FDA and CFDA, to provide a simple but dependable method for ADR signal detection and drug classification, as well as an auxiliary judgement basis for experts deciding on the status change of Rx drugs to OTC drugs. They propose that more ML or DL categorization algorithms be tested in the future and that computational complexity be factored into the comparison process. Kuenzi et al. (2020) built DrugCell, an interpretable DL algorithm of personal cancer cells based on the reactions of 1235 tumor cell lines to 684 drugs. Genotypes of cancer cause conditions in cellular systems combined with medication composition to forecast therapeutic outcome while also learning the molecular mechanisms underlying the response. Predictions made by DrugCell in cell lines are precise and help to categorize clinical outcomes. The study of DrugCell processes results in the development of medication combinations with synergistic effects, which we test using combinatorial CRISPR, in vitro drug–drug screening, and xenografts generated from patients. DrugCell is a step-by-step guide to building interpretable predictive medicine models.

Artificial Neural Networks (ANNs) that operate on graphs as inputs are known as Graph Neural Networks (GNNs). Deep GNNs were recently employed for learning representations of low-dimensional biomolecular networks (Hamilton 2020; Wu et al. 2020). Ahmed et al. (2020) used two separate GNN methods to develop a GNN using GE and a network of genes that are expressed together. This is a network that depicts the relationship between gene pairs' expression.

The CNN is one of the neural network models adopted for drug response prediction. The CNN has been actively used for image, video, text, and sound data due to its strong ability to preserve the local structure of data and learn hierarchies of features. In 2021, several methods had been developed for drug response prediction, each of which utilizes different input data for prediction (Baptista et al. 2021).

Nguyen et al. (2021) proposed a method to predict drug response called GraphDRP, which integrates two subnetworks for drug and cell line features, like CNN in Liu et al. (2019b) and Qiu et al. (2021). Gene expression data from cancer cell lines and medication response data, the author finds predictor genes for medications of interest and provides a reliable and accurate drug response prediction model. Using the Pearson correlation coefficient, they employed the ElasticNet regression model to predict drug response and fine-tune gene selection after pre-selecting genes. They ran a regression on each drug twice, once using the IC50 and once with the area under the curve (AUC), to obtain a more trustworthy collection of predictor genes (or activity area). The Pearson correlation coefficient for each of the 12 medicines they examined was greater than 0.6. With 17-AAG, IC50 has the highest Pearson correlation coefficient of 0.811.

In contrast, AUC has the highest Pearson correlation coefficient of 0.81. Even though the model developed in this study has excellent predictive performance for GDSC, it still has certain flaws. First, the cancer cell line's properties may differ significantly from those of in vivo malignancies, and it must be determined whether this will be advantageous in a clinical trial. Second, they primarily use gene expression data to predict drug response. While drug response is influenced by structural changes such as gene mutations, it is also influenced by gene expression levels. To improve the prediction capacity of the model, more research is needed to use such data and integrate it into the model.

In 2022, Ren et al. (2022) suggested a graph regularized matrix factorization based on deep learning (DeepGRMF), which uses a variety of information, including information on drug chemical composition, their effects on cell biology signaling mechanisms, and the conditions of cancer cells, to integrate neural networks, graph models, and matrix-factorization approaches to forecast cell response to medications. DeepGRMF trains drug embeddings so that drugs in the embedding space with similar structures and action mechanisms, (MOAs) are intimately linked. DeepGRMF learns the same representation embeddings for cells, allowing cells with similar biological states and pharmacological reactions to be linked. The Cancer Cell Line Encyclopedia (CCLE) and On the Genomics of Drug Sensitivity in Cancer (GDSC) datasets, DeepGRMF outperforms competing models in prediction performance. In the Cancer Genome Atlas (TCGA) dataset, the suggested model might anticipate the effectiveness of a treatment plan on lung cancer patients' outcomes. The limited expressiveness of our VAE-based chemical structure representation may explain why new cell line prediction outperforms innovative drug sensitivity prediction in terms of accuracy. A family of neural graph networks has recently been shown to depict better chemical structures that can be investigated in the future. Pouryahya et al. (2022) proposed a new network-based clustering approach for predicting medication response based on OMT theory. Gene-expression profiles and cheminformatic drug characteristics were used to cluster cell lines and medicines, and data networks were used to represent the data. Then, RF model was used regarding each pair of cell-line drug clusters. by comparison, prediction-clustered based models regarding the homogenous data are anticipated to enhance drug sensitivity and precise forecasting and biological interpretability.

### Drug–drug interactions (DDIs) side effect prediction using DL

Drugs are chemical compounds consumed by people and interact with protein targets to create a change. The drugs may alter the human body positively or negatively. Drug side effects are the undesirable alterations medications cause in the human body. These adverse effects might range from moderate headaches to life-threatening reactions like cardiac arrest, malignancy, and death. They differ depending on the person's age, gender, stage of sickness, and other factors (Kuijper et al. 2019). In the laboratory, to determine whether the medications have any unfavorable side effects, several tests are conducted on them. However, these examinations are both pricey and additionally lengthy. Recently, many computational algorithms for detecting medication adverse effects have been created. Computational methodologies are replacing laboratory experiments.

**Fig. 12:**
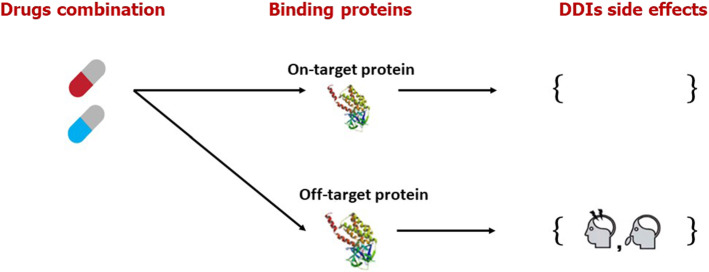
Drug binding with proteins and DDI side effects

On the other hand, these methods do not provide adequate data to predict drug–drug interactions (DDIs). The phenomenon of DDIs is discussed in Fig. 12. The desired effects of a drug resulting from its interaction with the intended target and the unfavorable repercussions emerging from drug interactions with off targets make up a drug's entire reaction on the human body (undesirable effects). Even though A medication has a strong affinity for binding to one target, it binds to several proteins as well with varied affinities, which might cause adverse consequences (Liu et al. 2021). Predicting DDIs can assist in reducing the likelihood of adverse reactions and optimizing the medication development and post-market monitoring processes (Arshed et al. 2022). Side effects of DDIs are often regarded as the leading cause of drug failure in pharmacological development. When drugs have major side effects, the market is quickly removed from them. As a result, predicting side effects is a fundamental requirement in the drug discovery process to keep drug development costs and timelines in check and launch a beneficial drug in terms of patient health recovery.

Furthermore, the average drug research and development cost is $2.6 billion (Liu et al. 2019). As a result, determining the possibility of negative consequences is important for lowering the expense and risk of medication development. The researchers use various computer tools to speed up the process. In pharmacology and clinical application, DDI prediction is a difficult topic, and correctly detecting possible DDIs in clinical studies is crucial for patients and the public. Researchers have recently produced a series of successes utilizing deep learning as an AI technique to predict DDIs by using drug structural properties and graph theory (Han et al. 2022). AI successfully detected potential drug interactions, allowing doctors to make informed decisions before prescribing prescription combinations to patients with complex or numerous conditions (Fokoue et al. 2016).

Therefore, this section comprehensively reviews the researchers' most popular DL algorithms to predict DDIs.

In 2016, Tiresias is a framework proposed by Achille Fokoue et al. (2017) for discovering DDIs. The Tiresias framework uses a large amount of drug-related data as input to generate DDI predictions. The detection of the DDI approach begins using input data that has been semantically integrated, resulting in a knowledge network that represents drug properties and interactions using additional components like enzymes, chemical structures, and routes. Numerous similarity metrics between all pharmacological categories were determined using a knowledge graph in a scalable and distributed setting. To forecast the DDIs, a large-scale logistic regression prediction model employs calculated similarity metrics. According to the findings, the Tiresias framework was proven to help identify new interactions between currently available medications and freshly designed and existing drugs. The suggested Tiresias model's necessity for big, scaled medication information was negative, resulting in the developed model's high cost.

In 2017, Reza et al. (2017) developed a computational technique for predicting DDIs based on functional similarities among all medicines. Several major biological aspects were used to create the suggested model: carriers, enzymes, transporters, and targets (CETT). The suggested approach was implemented on 2189 approved medications, for which the associated CETTs were obtained, and binary vectors to find the DDIs were created. Two million three hundred ninety-four thousand seven hundred sixty-seven potential drug–drug interactions were assessed, with over 250,000 unidentified possible DDIs discovered. Inner product-based similarity measures (IPSMs) offered good values predicted for detecting DDIs among the several similarity measures used. The lack of pharmacological data was a key flaw in this strategy, which resulted in the erroneous detection of all potential pairs of DDIs.

In 2018, Ryu et al. (2018) proposed a model that predicts more DDI kinds using the drug's chemical structures as inputs and applied multi-task learning to DDI type prediction in the same vein Decagon (Zitnik et al. 2018) models polypharmacy side effects using a relational GNN. To comprehend the representations of intricate nonlinear pharmacological interactions, Chu et al. (2018) utilized an auto-encoder for factoring. To predict DDIs, Liu et al. (2019c) presented the DDI-MDAE based on shared latent representation, a multimodal deep auto-encoder. Recently, interest in employing graph neural networks (GNNs) to forecast DDI has increased. Distinct aggregation algorithms lead to different versions of GNNs to efficiently assemble the vectors of its neighbors’ feature vectors (Asada et al. 2018) uses a convolutional graph network (GCN) to encode the molecular structures to extract DDIs from text. Furthermore, Ma et al. (2018) has incorporated attentive Multiview graph auto-encoders into a coherent model.

Chen (2018) devised a model for predicting Adverse Drug Reactions (ADR). SVM, LR, RF, and GBT were all used in the predictive model. The DEMO dataset, which contains properties such as the patient's age, weight, and sex, and the DRUG dataset, which includes features such as the drug's name, role, and dosage, were employed in this model. Males make up 46% of the sample, while females make up 54%. The developed model had a fair forecasting accuracy for a representative sample set. Furthermore, the outputs revealed that the suggested model is only accurate for a significant number of datasets.

To anticipate the possible DDI, Kastrin et al. (2018) employed statistical learning approaches. The DDI was depicted as a complex network, with nodes representing medications and links representing their potential interactions. On networks of DDIs, the procedure for predicting links was represented as a binary classification job. A big DDI database was picked randomly to forecast. Several supervised and unsupervised ML approaches, such as SVM, classification tree, boosting, and RF, are applied for edge prediction in various DDIs. Compared to unsupervised techniques, the supervised link prediction strategy generated encouraging results. To detect the link between the pharmaceuticals, The proposed method necessitates Unified Medical Language System (UMLS) filtering, which provided a dilemma for the scientists. Furthermore, the suggested system only considers fixed network snapshots, which is problematic for DDI's system because It's a fluid system.

In 2019, Lee et al. (2019) proposed a deep learning system for accurately forecasting the results of DDIs. To learn more about the pharmacological effects of a variety of DDIs, an assortment of auto-encoders and a deep feed-forward neural network was employed in the suggested method that were honed utilizing a mix of well-known techniques. The results revealed that using SSP alone improves GSP and TSP prediction accuracy, and the autoencoder is more powerful than PCA at reducing profile features. In addition, the model outperformed existing approaches and included numerous novel DDIs relevant to the current study Yue et al. (2020) combines numerous graphs embedding methods for the DDI job, while models DDI as link prediction with the help of a knowledge graph (Karim et al. 2019). There's also a system for co-attention (Andreea and Huang 2019), which presented a deep learning model based solely on side-effect data and molecular drug structure. CASTER in Huang et al. (2020) also based on drug chemical structures, develops a framework for dictionary learning to anticipate DDIs (Chu et al. 2019) and proposes using semi-supervised learning to extract meaningful information for DDI prediction in both labeled and unlabeled drug data. Shtar et al. (2019) used a mix of computational techniques to predict medication interactions, including artificial neural networks and graph node factor propagation methods such as adjacency matrix factorization (AMF) and adjacency matrix factorization with propagation (AMFP). The Drug-bank database was used to train the model, containing 1142 medications and 45,297 drug drugs. With 1442 drugs and 248,146 drug–drug interactions, the trained model was tested from the drug bank's most recent version. AMF and AMFP were also used to develop an ensemble-based classifier, and the outcomes were assessed using the receiver operating characteristic (ROC) curve. The findings revealed that the suggested a classifier that uses an ensemble delivers important drug development data and noisy data for drug prescription. In addition, drug embedding, which was developed during the training of models utilizing interaction networks, has been made available. To anticipate adverse drug events caused by DDIs, Hou et al. (2019) suggested a deep neural network architecture model. The suggested model is based on a database of 5000 medication codes obtained from Drug Bank. Using the computed features, it discovers 80 different types of DDIs. Tensor Flow-GPU was also used to create the model, which takes 4432 drug characteristics as input.

Medicines for inflammatory bowel disease (IBD) can predict how they will react; the trained model has an accuracy of 88 percent. The findings also revealed that the model performs best when many datasets are used. Detecting negative effects of drugs with a DNN Model was proposed by Wang et al. (2019). The model predicts ADRs by using synthetic, biological, and biomedical knowledge of drugs. Drug data from SIDER databases was also incorporated into the model. The proposed system's performance was improved by distributing. Using a word-embedding approach, determine the association between medications using the target drug representations in a vector space. The suggested system's fundamental flaw was that it only worked well with ordinary SIDER databases.

In 2020, numerous AI-based methods were developed for DDI event prediction, including evaluating chemical structural similarity using neural graph networks (Huang et al. 2020). Attempts to forecast DDI utilizing different data sources have also been made, such as leveraging similarity features to create pharmacological features for the DDI job predicting occurrences (Deng et al. 2020).

With the help of word embeddings, part-of-speech tags, and distance embeddings. Bai et al. (2020) suggested a deep learning technique that executes the DDI extraction task and supports the drug development cycle and drug repurposing. According to experimental data, the technique can better avoid instance misclassifications with minimal pre-processing. Moreover, the model employs an attention technique to emphasize the significance of each hidden state in the Bi-LSTM layers.

A tool for extracting features regarding a graph convolutional network (GCN) and a predictor based on a DNN. Feng et al. (2020) suggested DPDDI, an effective and robust approach for predicting potential DDIs by utilizing data from the DDI network lacking a thought of drug characteristics (i.e., drug chemical and biological properties). The proposed DPDDI is a useful tool for forecasting DDIs. It should benefit from other DDI-related circumstances, such as recognizing unanticipated side effects and guiding drug combinations. The disadvantage of this paradigm is that it ignores drug characteristics.

Zaikis and Vlahavas (2020), by developing a bi-level network with a more advanced level reflecting the network of biological entities' interactions, suggested a multi-level GNN framework for predicting biological entity links. Lower levels, however, reflect individual biological entities such as drugs and proteins, although the proposed model's accuracy needs to be enhanced.

In 2021, To overcome the DDI prediction, Lin et al. (2021) suggested an end-to-end system called Knowledge Graph Neural Network (KGNN). KGNN expands the use of spatial GNN algorithms to the knowledge graph by selectively various aggregators of neighborhood data, allowing it to learn the knowledge graph's topological structural information, semantic relations, and the neighborhood of drugs and drug-related entities. Medical risks are reduced when numerous medications are used correctly, and drug synergy advantages are maximized. For multi-typed DDI pharmacological effect prediction, Yue et al. (2021) used knowledge graph summarization. Lyu et al. (2021) also introduced a Multimodal Deep Neural Network (MDNN) framework for DDI event prediction. On the drug knowledge graph, a graph neural network was used, MDNN effectively utilizes topological information and semantic relations. MDNN additionally uses joint representation structure information, and heterogeneous traits are studied, which successfully investigates the multimodal data's complementarity across modes. Karim et al. (2019) built a knowledge graph that used CNN and LSTM models to extract local and global pharmacological properties across the network. DANN-DDI is a deep attention neural network framework proposed by Liu et al. (2021). To anticipate unknown DDIs, it carefully incorporates different pharmacological properties (Chun and Yi-Ping Phoebe 2021) and developed a deep hybrid learning (DL) model to provide a descriptive forecasting of pharmacological adverse reactions. It was one of the initial hybrid DL models through conception models that could be interpreted. The model includes a graph CNN through conception models to improve the learning efficiency of chemical drug properties and bidirectional long short-term memory (BiLSTM) recurrent neural networks to link drug structure to adverse effects. After concatenating the outputs of the two networks (GCNN and BiLSTM), a fully connected network is utilized to forecast pharmacological adverse reactions. Regardless of the classification threshold, the model obtains an AUC of 0.846. It has a 0.925 precision score. Even though a tiny drug data set was used for adverse drug response (ADR) prediction, the Bilingual Evaluation Understudy (BLEU) concluded results were 0.973, 0.938, 0.927, and 0.318, indicating considerable achievements. Furthermore, the model can correctly form words to explain pharmacological adverse reactions and link them to the drug's name and molecular structure. The projected drug structure and ADR relationship will guide safety pharmacology research at the preclinical stage and make ADR detection easier early in the drug development process. It can also aid in the detection of unknown ADRs in existing medications. DDI extraction using a deep neural network model from medical literature was proposed by Mohsen and Hossein (). This model employs an innovative approach of attracting attention to improve the separation of essential words from other terms based on word similarity and location concerning candidate medications. Before recognizing the type of DDIs, this method calculates the results of a bi-directional long short-term memory (Bi-LSTM) model's attention weights in the deep network architecture. On the standard DDI Extraction 2013 dataset, the proposed approach was tested. According to the findings of the experiments, they were able to get an F1-Score of 78.30, which is comparable to the greatest outcomes for stated existing approaches.

In 2022, Pietro et al. (2022) introduced DruGNN, a GNN-based technique for predicting DDI side effects. Each DDI corresponds to a class in the prediction, a multi-class, multi-label node classification issue. To forecast the side effects of novel pharmaceuticals, they use a combination inductive-transudative learning system that takes advantage of drug and gene traits (induction path) and knowledge of known drug side effects (transduction path). The entire procedure is adaptable because the base for machine learning can still be used if the graph dataset is enlarged to include more node properties and associations. Zhang et al. (2022) proposed CNN-DDI, a new semi-supervised algorithm for predicting DDIs that uses a CNN architecture. They first extracted interaction features from pharmacological categories, targets, pathways, and enzymes as feature vectors. They then suggested a novel convolution neural network as a predictor of DDIs-related events based on feature representation. Five convolutional layers, two full-connected layers, and a CNN-based SoftMax layer make up the predictor. The results reveal that CNN-DDI superior to other cutting-edge techniques, but it takes longer to complete (Jing et al. 2022) presented DTSyn. This unique dual-transformer-based approach can select probable cancer medication combinations. It uses a multi-head attention technique to extract chemical substructure-gene, chemical-chemical, and chemical-cell-line connections. DTSyn is the initial model that incorporates two transformer blocks to extract linkages between interactions between genes, drugs, and cell lines, allowing a better understanding of drug action processes. Despite DTSyn's excellent performance, it was discovered that balanced accuracy on independent data sets is still limited. Collecting more training data is expected to solve the problem. Another issue is that the fine-granularity transformer was only trained on 978 signature genes, which could result in some chemical-target interactions being lost.

Furthermore, DTSyn used expression data as the only cell line attributes. To fully represent the cell line, additional omics data may be added going forward, including methylation and genetic data. He et al. (2022) proposed MFFGNN, a new end-to-end learning framework for DDI forecasting that can effectively combine information from molecular drug diagrams, SMILES sequences, and DDI graphs. The MFFGNN model used the molecular graph feature extraction module to extract global and local features from molecular graphs.

They run thorough tests on a variety of real-world datasets. The MFFGNN model routinely beats further cutting-edge models, according to the findings. Furthermore, the module for multi-type feature fusion configures the gating mechanism to limit the amount of neighborhood data provided to the node.

### Drug–drug similarity prediction using DL

Drug similarity studies presume that medications with comparable pharmacological qualities have similar activation mechanisms, and side effects are used to treat problems like each other (Brown 2017; Zeng et al. 2019).

**Fig. 13:**
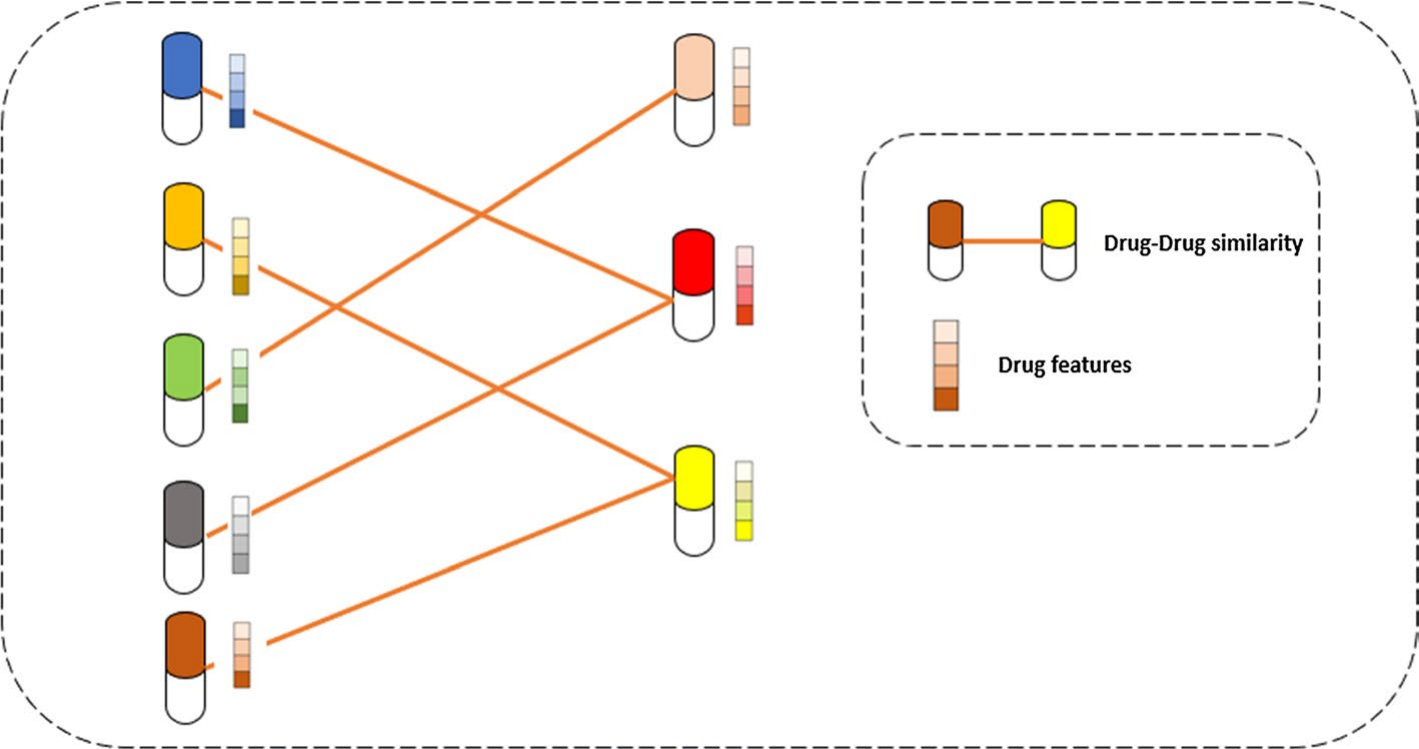
Drug–drug similarity main idea

The drug-pharmacological similarity is critical for various purposes, including identifying drug targets, predicting side effects, predicting drug–drug interactions, and repositioning drugs. Features of the chemical structure (Lu et al. 2017; O’Boyle 2016), protein targets (Vilar 2016; Wang et al. 2014), side-effect profiles (Campillos et al. 2008; Tatonetti et al. 2012), and gene expression profiles (Iorio et al. 2010) provide a multi-perspective viewpoint for forecasting medications that are similar and can correct for data gaps in different data sources and offer fresh perspectives on drug repositioning and other uses. The main idea of drug–drug similarity is presented in Fig. 13. The vector represents the drug features, and the links reflect the similarity between the two drugs.

#### Drug similarity measures

The similarity estimations are calculated based on chemical structure, target protein sequence-based, target protein functional, and drug-induced pathway similarities.

##### The similarity in chemical structure

DrugBank (2019) provides tiny molecule medicine chemical structures in SDF molecular format. Invalid SDFs can be recognized and eliminated, such as those with a NA value or fewer than three columns in atom or bond blocks. For valid compounds, atom pair descriptors can be computed, pairwise comparison of compounds, δ_*c*_ (*di*, *dj*), was evaluated using atom pairs using the Tanimoto coefficient, which is defined as the number of atom pairs in each fraction shared by two different compounds divided by their union (Eq. 1).

1 $$ \delta_{c} \left( {di,dj} \right) = {{\left| {AP_{i} \cap AP_{j} } \right|} \mathord{\left/ {\vphantom {{\left| {AP_{i} \cap AP_{j} } \right|} {\left| {AP_{i} \cup AP_{j} } \right|}}} \right. \kern-\nulldelimiterspace} {\left| {AP_{i} \cup AP_{j} } \right|}} $$

where AP_i_ and AP_j_ are atom pairs from pharmaceuticals d_i_ and dj, respectively, the numerator is the total number of atom pairs in both compounds, while the denominator is the number of common atom pairs in both compounds.

##### Target protein sequence-based similarity

DrugBank provides all small molecule drugs have target sequences in FASTA format. The basic Needleman-Wunsch et al. (1970) dynamic programming approach for global alignment can be used to compare pairwise protein sequences. The proportion of pairwise sequence identity (Raghava 2006) can be represented as the corresponding sequence similarity. Equation 2 was used to calculate drug–drug similarity based on target sequence similarities:

2 $$ \delta_{{\text{t}}} { }\left( {{\text{d}}_{{\text{i}}} ,{\text{d}}_{{\text{j}}} } \right) = \frac{{\left( {\mathop \sum \nolimits_{{{\text{x}} \in {\text{T}}_{{\text{i}}} }} {\text{max}}_{{\forall {\text{y}} \in {\text{T}}_{{\text{j}}} }} \left\{ {{\text{S}}\left( {{\text{x}},{\text{y}}} \right)} \right\} + { }\mathop \sum \nolimits_{{{\text{x}} \in {\text{T}}_{{\text{j}}} }} {\text{max}}_{{\forall {\text{x}} \in {\text{T}}_{{\text{i}}} }} \left\{ {{\text{S}}\left( {{\text{y}},{\text{x}}} \right)} \right\}} \right)}}{{\left( {\left| {{\text{T}}_{{\text{i}}} } \right|{* }\left| {{\text{T}}_{{\text{j}}} } \right|} \right)}} $$

where δ*t* (*di*, *dj*) denotes target-based similarity between medicines di and dj. Drugs di target a group of proteins known as Ti. Tj is a set of proteins that pharmaceuticals dj target and S(x,y) is a similarity metric based on symmetric sequences between two targeted proteins, x $\in$ Ti and y $\in$ Tj. Overall, Eq. 2 calculates the average of the best matches, wherein each first medicine's target is only connected to the second medicine's most comparable phrase, and vice versa.

##### Target protein functional similarity

Protein targets that are overrepresented by comparable biological functions and have similar sequences imply shared pharmacological mechanisms and downstream effects (Passi et al. 2018). As a result, each protein has a set of Gene Ontology (GO) concepts from all three categories associated with it, such as cellular components (CC), molecular functions (MF), and biological processes (BP). We filtered out GO keywords that were either very specialized (with 15 linked genes) or very general (with 100 genes). DrugBank (2019) provided the Human Protein–Protein Interaction (PPI) network. Wang et al. (2007) proposed leveraging the topology of the GO graph structure to determine the semantic similarity of their linked GO terms, which was used to determine how functionally comparable two drugs are, such as δ_f_ (d_i_, d_j_). Using a best-match average technique, any two GO keywords are compared for pairwise semantic similarity connected with di and d_j_ were aggregated into a single semantic similarity measure and presented into a final similarity matrix.

##### Drug-induced pathway similarity

A medication pair that triggers similar pathways or overlaps shows that the drugs' mechanisms of action are similar, which is useful information for drug similarities and repositioning research (Zeng et al. 2015). Kanehisa and Goto (2000) was used to find the pathways activated by each small molecule medication. Using dice similarity, the similarity in pairs of any two options was calculated based on their constituent genes' closeness. After that, a pathway-based similarity score was calculated for each medication pair d_i_ and d_j_, i.e., δ_*p*_ (*d*_*i*_, *d*_*j*_), was calculated using Eq. 3:

3 $$ \delta_{p} \left( {d_{i} ,d_{j} } \right) = \mathop {\max }\limits_{{\forall x \in P_{i} ,\forall y \in P_{j} }} \left\{ {DSC\left( {x,y} \right)} \right\}, $$

where *P*_*i*_ and *P*_*j*_ are a group of drug-induced pathways *d*_*i*_ and *d*_*j*_, respectively; *x* and *y* are two paths represented by a group of genes that make up their constituents, and $DSC\left( {x,y} \right) = {{{2}\left| {x \cap y} \right|} \mathord{\left/ {\vphantom {{{2}\left| {x \cap y} \right|} {\left( {\left| x \right| + \left| y \right|} \right)}}} \right. \kern-\nulldelimiterspace} {\left( {\left| x \right| + \left| y \right|} \right)}}$ is the probability of a pair of dice matching, this determines how much the two trajectories overlap. When no gene is shared by any two pathways produced by the comparing drug pair, the similarity is set to 0.0. Overall, Eq. 3 implies that if two medications stimulate one or more identical pathways, the maximum pathway-based similarity will be achieved (s).

#### DL for drug similarity prediction

Wang et al. (2019) introduced a gated recurrent units (GRUs) model that employs similarity to predict drug–disease interactions. In this approach, CDK turned the SMILES into 2D chemical fingerprints, and the Jaccard score of the 2D chemical fingerprints was used to compare the two medicines. This section comprehensively reviews the researchers' most popular DL algorithms to predict drug similarity.

Hirohara et al. (2018) employed a CNN to learn molecular representation. The network is given the molecule's SMILES notation as input to feed into the convolutional layers in this scenario. The TOX 21 dataset was used.

To conduct similarity analysis, Cheng et al. (2019) used the Anatomical Therapeutic Chemical (ATC) based on the drug ATC classification systems and code-based commonalities of drug pairs. The authors created interaction networks, performed drug pair similarity analyses, and developed a network-based methodology for identifying clinically effective treatment combinations for a specific condition.

Xin et al. (2016) presented a Ranking-based k-Nearest Neighbour (Re-KNN) technique for medication repositioning. The method's key feature combines the Ranking SVM (Support Vector Machine) algorithm and the traditional KNN algorithm. Chemical structural similarity, target-based similarity, side-effect similarity, and topological similarity are the types of similarity computation methodologies they used. The Tanimoto score was then used to determine the similarity between the two profiles.

Seo et al. (2020) proposed an approach that combined drug–drug interactions from DrugBank, network-based drug–drug interactions, polymorphisms in a single nucleotide, and anatomical hierarchy of side effects, as well as indications, targets, and chemical structures.

Zeng et al. (2019) developed an assessment of clinical drug–drug similarity derived from data from the clinic and used EHRs to analyse and establish drug–diagnosis connections. Using the Bonferroni adjusted hypergeometric P value, they created connections between drugs and diagnoses in an EMR dataset. The distances between medications were assessed using the Jaccard similarity coefficient to form drug clusters, and a k-means algorithm was devised.

Dai et al. (2020) reviewed, summarized representative methods, and discussed applications of patient similarity. The authors talked about the values and applications of patient similarity networks. Also, they discussed the ways to measure similarity or distance between each pair of patients and classified it into unsupervised, supervised, and semi-supervised.

Yan et al. (2019) created BiRWDDA, a new computational methodology for medication repositioning that combines bi-random walk and various similarity measures to uncover potential correlations between diseases and pharmaceuticals. First drug and disease–disease similarities are assessed to identify optimal drug and disease similarities. The information entropy is evaluated between the similarity of medicine and disease to determine the right similarities. Four drug–drug similarity metrics and three disease–disease similarity measurements were calculated depending on some drug- and disease-related characteristics to create a heterogeneous network. The drug's protein sequence information, the extracted drug interaction from DrugBank then utilized the Jaccard score to determine this similarity, the chemical structure, derived canonical SMILES from DrugBank, and the side effect, respectively the four drug–drug similarities.

Yi et al. (2021) constructed the model of a deep gated recurrent unit to foresee drug–disease interactions that likely employ a wide range of similarity metrics and a kernel with a Gaussian interaction profile. Based on their chemical fingerprints, the similarity measure is utilized to detect a distinguishing trait in medications. Meanwhile, based on established disease–disease relationships, the Gaussian interactions profile kernel is used to derive efficient disease features. After that, a model with a deep gated recurrent cycle is created to anticipate drug-disease interactions that could occur. The outputs of the experiments showed that the suggested algorithm could be used to anticipate novel drug indications or disease treatments and speed up drug repositioning and associated drug research and discovery.

To forecast DDIs, Yan et al. (2022) suggested a semi-supervised learning technique (DDI-IS-SL). DDI-IS-SL uses the cosine similarity method to calculate drug feature similarity by combining chemical, biological, and phenotypic data. Drug chemical structures, drug–target interactions, drug enzymes, drug transporters, drug routes, drug indications, drug side effects, harmful effects of drug discontinuation, and DDIs that have been identified are all included in the integrated drug information.

Heba et al. (2021) used DrugBank to develop a machine learning framework based on similarities called "SMDIP" (Similarity-based ML for Drug Interaction Prediction), where they calculated drug–drug similarity utilizing a Russell–Rao metric for the biological and structural data that is currently accessible on DrugBank to represent the limited feature area. The DDI classification is carried out using logistic regression, emphasizing finding the main predictors of similarity. The DDI key features are subjected to six machine learning models (NB: naive Bayes; LR: logistic regression; KNN: k-nearest neighbours; ANN: neural network; RFC: random forest classifier; SVM: support vector machine).

For large-scale DDI prediction, Vilar et al. (2014) provided a procedure combining five similar drug fingerprints (Two-dimensional structural fingerprints, fingerprinting of interaction profiles, fingerprints of the target profile, Fingerprints of ADE profiles, and pharmacophoric techniques in three dimensions).

Song et al. (2022) used similarity theory and a convolutional neural network to create global structural similarity characteristics. They employed a transformer to extract and produce local chemical sub-structure semantic characteristics for drugs and proteins. To create drug and protein global structural similarity characteristics, The Tanimoto coefficient, Levenshtein distance, and CNN are all utilized in this study.

## Benchmark datasets and databases

Drug development or discovery has been based on a range of direct and indirect data sources and has regularly demonstrated strong predictive capability in finding confirmed repositioning candidates and other applications for computer-aided drug design. This section reviews the most important and available benchmark datasets and databases used in the drug discovery problem and which the researchers may need according to each problem category. Thirty-five datasets are summarized in Table 3.

## Evaluation metrics

Performance measures are required for evaluating machine learning models (Benedek et al. 2021). The measures serve as a tool for comparing different techniques. They aid in comparing many approaches to identify the best one for execution. This section describes the many metrics defined for the four categories of drug discovery difficulties below.

Table 4 shows the metrics employed in drug discovery problems—understanding the metrics aids in assessing the effectiveness of various prediction systems. True positives (TP) are drug side effects that have been recognized appropriately, False positives (FP) are adverse pharmacological effects that aren't present but were detected by the model, and True negatives (TN) are pharmacological side effects that do not exist but that the model failed to detect. False negatives (FN) are adverse pharmacological effects the model did not predict.

**Table 4:** The important metrics for drug discovery problems

Metrics	Description	Equations
Accuracy	It aids in determining the total number of side effects appropriately identified using the proposed technique	Accuracy = (TP + TN)/(TP + TN + FP + FN)
Sensitivity and specificity	The sensitivity metric aids in the identification of accurately identified positive samples. Specificity, on the other hand, detects the correctly established negative samples	Sensitivity = TP/(TP + FN), Specificity = TN/(TN + FP)
Precision	The precision metric essentially identifies the relevant cases among all the expected events. As a result, it can be defined as true side effects resulting from all of a given technique's expected adverse effects	Precision = TP/(TP + FP)
Recall	The total number of real-life situations that have been discovered out of a total of accurate occurrences is referred to as recall	Recall = TP/(TP + FN)
F-measure	This statistic is used to assess a technique's accuracy. Precision and recall's harmonic mean can be defined as this	F-measure = 2 × ((precision × recall)/(precision + recall))
ROC curve and AUC score	The receiving operator characteristic (ROC) curve is used to evaluate a model's performance across a variety of criterion values. It contrasts the ratio of true positives to false positives at various threshold values The area under the ROC curve is measured by the AUC value. It aids in the model's proper evaluation because it is scale invariant and regardless of the value of the threshold	
PR curve and AUPR value	Although AUC cure has been used to evaluate the efficacy of several pharmacological Prediction models for side effects, most field-specific data sets are unbalanced, and the methodologies may exaggerate the AUC scores. As a result, the precision–recall (PR) curve has been employed to assess performance. At various threshold points, it compares the value of precision to recall. In addition, The AUPR value is a summary of the PR value over a wide range of thresholds	
Rate of predictions	The percentage of accurate projections and the frequency with which targets are missed have also been used to evaluate performance in some techniques	Rate of correct predictions = number of predictions/total number of experimentally known instances
Computational cost	The computational cost of a technology is defined as the overall time it takes to implement it on a given system. In most cases, it is measured in seconds	
Multilabel evaluation metrics	Some research regard side effect prediction to be a multilabel classification problem, and thus use multilabel evaluation measures to assess performance. Hammering loss, one error, coverage, and ranking loss are some of the most widely utilized measures	
Binding Affinity Score	Several assessment indicators can be used to examine it such as mean square error (MSE) and root mean square error (RMSE). These standards have been applied to rate the quality of the predictive QSAR model. The average squared difference between the predicted and actual scores for binding affinity is known as the MSE.The RMSE is the squaredMSE, as the name implies	

## Drug dosing optimization

Drugs are vital to human health and choosing the proper treatment and dose for the right patient is a constant problem for clinicians. Even when taken as studied and prescribed, drugs have adverse impact profiles with varying response rates. As a result, all medications must be well-managed, especially those utilized in treating critical ailments or with a tight exposure window between efficacy and toxicity. Clinicians follow typical guidelines for the first dosage, which is not always optimal or secure for every patient, especially if the medicine no longer is evaluated in various dosages for various patient types. Precision dosage can revolutionize by increasing perks in health care while reducing drug therapy risks. While precise dosing will probably influence some pharmaceuticals significantly, perhaps not essential or practical to apply to all drugs or therapeutic classes. As a result, recognizing the characteristics that make medications suitable for precision dosage targets will aid in directing resources to where they'll have the most impact. Precision-dosing meds with a high priority and therapeutic classes could be crucial in achieving increased health care performance, safety, and cost-effectiveness (Tyson et al. 2020).

Due to standard, fixed dosing procedures or gaps in knowledge, imprecise drug dosing in specific subpopulations increases the risk of potentiating adverse effects due to supratherapeutic or subtherapeutic concentrations (Watanabe et al. 2018). Currently, the Food and Medicine Administration (FDA) simply requires a drug to be statistically better than a non-inferior to placebo of the existing treatment standard. This does not guarantee that the medicine will benefit most patients in clinical trials, especially if malignancies treatment can be tough, like diffuse intrinsic pontine glioma (DIPG) and unresectable meningioma, where rates of therapy response can be exceedingly low (Fleischhack et al. 2019).

There are essential aspects for dose optimization (https://friendsofcancerresearch.org/wpcontent/uploads/Optimizing_Dosing_in_Oncology_Drug_Development.pdf) that vary based on the product, the target population, and the available data to find the most effective dose, which varies based on the product, the target population, and the available data:

- Therapeutic properties: Drug features such as small molecule vs. large molecule and agonist vs. antagonist impact how drugs interact with the body regarding safety and efficacy. The therapeutic characteristics impact the first doses used in dose-finding studies and the procedures used to determine which doses should be used in registrational trials.
- Patient populations: Patient demographics vary depending on tumour kind, stage of disease, and comorbidities. Understanding how diverse factors influence the drug's efficacy may justify modifying the dose correspondingly, especially in the context of enlarged clinical trial populations.
- Supplemental versus original approval: Differences in disease features and patient demographics between tumour types and treatment settings, such as monotherapy versus combination therapy, must be considered when assessing whether additional dose exploration is required for a supplemental application. In cases when more dose exploration is required, the research design can include previous exposure-response knowledge from the initial approval.

## Drug discovery and XAI

The topic of XAI addresses one of the most serious flaws in ML and DL algorithms: model interpretability and explain ability. Understanding how and why a prediction is formed becomes increasingly crucial as algorithms grow more sophisticated and can forecast with greater accuracy. It would be impossible to trust the forecasts of real-world AI applications without interpretability and explain ability. Human-comprehensible explanations will increase system safety while encouraging trust and sustained acceptance of machine learning technologies (). XAI has been studied to circumvent the limitations of AI technologies due to their black-box nature. In contrast to making decisions and model justifications which may be provided by AI approaches like DL and XAI (Zhang et al. 2022). Attention has been attracted to XAI approaches (Lipton 2018; Murdoch et al. 2019) to compensate for the lack of interpretability of some ML models as well as to aid human decision-making and reasoning (Goebel et al. 2018). The purpose of presenting relevant explanations alongside mathematical models is to help students understand them better by (1) Making the decision-making process more transparent (Doshi-Velez and Kim 2017), (2) correct predictions should not be made for the wrong motives (Lapuschkin et al. 2019), (3) avoid biases and discrimination that are unjust or unethical (Miller 2019), and (4) close the gap between ML and other scientific disciplines. Effective XAI can also help scientists in navigating the scientific process (Goebel et al. 2018), enabling people to fine-tune their understanding and opinions on the process under inquiry (Chander et al. 2018). We hope to provide an overview of recent XAI drug discovery research in this section.

XAI has a place in drug development. While the precise definition of XAI is still up for controversy (Guidotti et al. 2018), the following characteristics of XAI are unquestionably beneficial in applications of drug design (Lipton 2018):

- Transparency is accomplished by understanding how the system came to a specific result.
- The explanation of why the model's response is suitable serves as justification. It is instructive to provide new information to human decision-makers.
- Determining the reliability of a prediction to estimate uncertainty.

The molecular explanation of pharmacological activity is already possible with XAI (Xu et al. 2017; Ciallella and Zhu 2019), as well as drug safety and organic synthesis planning (Dey et al. 2018). If It's working overtime, XAI will be important in processing and interpreting increasingly complex chemical data, as well as creating new pharmaceutical ideas, all while preventing human bias (Boobier et al. 2017). Application-specific XAI techniques are being developed to quickly reply to unique scientific issues relating to the Pathophysiology and biology of the human may be boosted by pressing drug discovery difficulties such as the coronavirus pandemic.

AI tools can increase their prediction performance by increasing model complexity. As a result, these models become opaque, with no clear grasp of how they operate. Because of this ambiguity, AI models are not generally utilized in important industries such as medical care. As a result, XAI focuses on understanding what goes into AI model prediction to meet the demand for transparency in AI tools. AI model interpretability approaches can be categorized depending on the algorithms used, a scale for interpreting, and the kind of information (Adadi and Mohammed 2018). Regarding the objectives of interpretability, approaches grouped as white-box model development, black-box model explanation, model fairness enhancement, and predictive sensitivity testing (Guidotti et al. 2018).

According to the gradient-based attribution technique (Simonyan et al. 2014), the network's input features are to blame for the forecast. Because this strategy is commonly employed when producing a DNN system's predictions, it may be a suitable solution for various black-box DNN models in DDI prediction (Quan, et al. 2016; Sun et al. 2018). In addition, DeepLIFT is a frequent strategy for implementing on top of DNN models that have been demonstrated to be superior to techniques based on gradients (Shrikumar et al. 2017). As opposed to that, the Guided Backpropagation model may be used to construct network architectures (Springenberg 2015). A convolutional layer with improved stride can be used instead of max pooling in CNN to deal with loss of precision. This method could be employed in CNN-based DDI prediction, as shown in Zeng et al. (2015).

Furthermore, in the Tao et al. (2016) was implemented neural networks that parse natural language. Using rationales, this method aimed to achieve the small pieces of input text. This method's design comprises two parts: a generator and an encoder that seek for text subsets that are closely connected to the predicted outcome. Because NLP-based models are used to extract DDIs (Quan et al. 2016), the above methods should be examined for usage in improving the model's clarity.

Aside from that, XAI has created methods for developing white-box models, including linear, decision tree, rule-based, and advanced but transparent models. However, these approaches are receiving less attention due to their weak ability to predict, particularly in the NLP-based sector, such as in the DDIs the job of extracting. Several ideas to address AI fairness have also been offered. Nonetheless, while extracting DDIs, only a small number of these scholarly studies looked at non-tabular data impartiality, such as text-based data. Many DDIs experiments used the word embedding method (Quan et al. 2016; Zhang 2020; Bolukbasi 2016). As a result, attempts to ensure fairness in DDI research should be considered more. To ensure the reliability of AI models, numerous methods also make an effort to examine the sensitivity of the models. Regarding their Adversarial Example-based Sensitivity Analysis, Zügner et al. (2018) used this model to explore graph-structured data. The technique looks at making changes to links between nodes or node properties to target node categorization models. Because graph-based methods are frequently utilized in DDIs research (Lin et al. 2021; Sun et al. 2020b), methods like those used in the previous study suggest that they might be used in a DDIs prediction model. In RNN, word embedding perturbations (Miyato et al. 1605) are also worth addressing. Significantly, the input reduction strategy utilized by Feng et al. (2018) to expose hypersensitivity in NLP models could be applied to DDI extraction studies. The DDIs study of Schwarz et al. (2021) attempted to provide model interpretability using Attention ratings derived at all levels of modeling in their DDIs study. The significance of similarity matrices to the vectors for medication depiction is determined using these scores, and drug properties that contribute to improved encoding are identified using these scores. This method makes use of data that travels through all tiers of the network.

Graph neural networks (GNNs) and their explain ability are rapidly evolving in the field of graph data. GNNExplainer in Ying et al. (2019) uses mask optimization to learn soft masks for edge and node attributes to elaborate on the forecasts. Soft masks have been initiated at random and regarded as trainable variables. After that, the masks are then combined in comparison to the first graph using multiplications on a per-element basis by GNNExplainer. After that, by enhancing the exchange of information between the forecasts from the first graph and the recently acquired graph, the masks are maximized. Even when various regularization terms, such as element-by-element entropy, motivate optimal disguises for stealth, the resulting Masks remain supple.

In addition, because the masks are tuned for each input graph separately, it’s possible that the explanations aren't comprehensive enough. To elaborate on the forecasts, PGExplainer (Luo et al. 2020) discovers approximated discrete edge masks. To forecast edge masks, it develops a mask predictor that is parameterized. It starts by concatenating node embeddings to get the embeddings for each edge in an input graph. The predictor then forecasts the chances of each edge being selected using the edge embeddings, that regarded as an evaluation of significance. The reparameterization approach is then used to sample the approximated discrete masks. Finally, the mutual information between the previous and new forecasts is optimized to train the mask predictor. GraphMask (Schlichtkrull et al. 2010) describes the relevance of edges in each GNN layer after the fact. It uses a classifier, like the PGExplainer, to forecast if an edge may be eliminated and does not impact the original predictions. A binary concrete distribution (Louizos et al. 1712) and a reparameterization method are used to roughly represent separate masks. The classifier is additionally trained by removing a term for a difference, which evaluates the difference between network predictions over the entire dataset. ZORRO (Thorben et al. 2021) employs discrete masks to pinpoint key input nodes and characteristics. A greedy method is used to choose nodes or node attributes from an input network. ZORRO chooses one node characteristic with the greatest fidelity score for each stage. The objective function, fidelity score, measures the degree of the recent forecasts resemble the model's original predictions by replacing the rest of the nodes/features with random noise values and repairing chosen nodes/features. The non-differentiable limitation of discrete masks is overcome because no training process is used.

Furthermore, ZORRO avoids the problem of "introduced evidence" by wearing protective masks. The greedy mask selection process, on the other hand, may result in optimal local explanations. Furthermore, because masks are generated for each graph separately, the explanations may lack a global understanding. Causal Screening (Xiang et al. 2021) investigates the attribution of causality to various edges in the input graph. It locates the explanatory subgraph's edge mask. The essential concept behind causal attribution is to look at how predictions change when an edge is added to the present explanatory subgraph, called the influence of causality. It examines the causal consequences of many edges at each step and selects one to include in the paragraph. It selects edges using the individual causal effect (ICE), which assesses the difference in information between parties after additional edges are introduced to the subgraph.

Causal Screening, like ZORRO, is a rapacious algorithm that generates undetectable masks without any prior training. As a result, it does not suffer due to the issue of the evidence presented. However, it is possible to lack worldwide comprehension and be caught in optimum local explanations. SubgraphX (Yuan et al. 2102) investigates deep graph model subgraph-level explanations. It uses the Monte Carlo Tree Search (MCTS) method (Silver et al. 2017) to effectively investigate various subgraphs by trimming nodes and choose the most significant subgraph from the search tree's leaves as the explanation for the prediction.

Furthermore, the Shapley values can be used to update the mask generation algorithm's objective function. Its produced subgraphs are more understandable by humans and suited for graph data than previous perturbation-based approaches. However, the computational cost is higher because the MCTS algorithm explores distinct subgraphs.

## Success stories about using DL in drug discovery

Big pharmaceutical companies have migrated toward AI as DL methodologies have advanced, abandoning conventional approaches to maximize patient and company profit. AstraZeneca is a multinational, science-driven, worldwide pharmaceutical company that has successfully used artificial intelligence in each stage of drug development, from virtual screening to clinical trials. They could comprehend current diseases better, identify new targets, plan clinical trials with higher quality, and speed up the entire process by incorporating AI into medical science. AstraZeneca's success is a shining illustration of how combining AI with medical science can yield incredible results. Their collaborations with other AI-based companies demonstrate their continual attempts to increase AI utilization. One such cooperation is with Ali Health, an Alibaba subsidiary that wants to provide AI-assisted screening and diagnosis systems in China (Nag et al. 2022).

SARS-CoV-2 virus outbreak placed many businesses under duress to develop the best medicine in the shortest amount of time feasible. These businesses have turned to employ AI in conjunction based on the data available to attain their goals. Below are some examples of firms that have been successful in identifying viable strategies to combat the COVID-19 virus because of their efforts.

Deargen, a South Korean startup, developed the MT-DTI (Molecule Transformer Drug Target Interaction Model), a DL-based drug-protein interaction prediction model. In this approach, the strength of an interaction between a drug and its target protein is predicted using simplified chemical sequences rather than 2D or 3D molecular structures. A critical protein on the COVID-19-causing virus SARS-CoV-2 is highly likely to bind to and inhibit the FDA-approved antiviral drug atazanavir, a therapy for HIV. It also discovered three more antivirals, as well as Remdesivir, a not-yet-approved medicine that is currently being studied in patients. Deagen's ability to uncover antivirals utilizing DL approaches is a significant step forward in pharmaceutical research, making it less time-consuming and more efficient. If such treatments are thoroughly evaluated, there is a good chance that we will be able to stop the epidemic in its tracks (Beck et al. 2020; Scudellari 2020).

Another example is Benevolent AI, a biotechnology company in London leverages medical information, AI, and machine learning to speed up health-related research. They've identified six medicines so far, one of which, Ruxolitinib, is claimed to be in clinical trials for COVID19 (Gatti et al. 2021). To find prospective medications that might impede the procedure for viral replication of SARS-CoV-2, The business has been utilizing a massive reservoir of information pertaining to medicine, together Utilizing data obtained from the scientific literature by their AI system and ML. They received FDA permission to use their planned Baricitinib medication in conjunction with Remdesivir, which resulted in a higher recovery rate for hospitalized COVID19 patients (Richardson et al. 2020).

Skin cancer is a form of cancer that is very frequent around the globe. As the rate at which skin cancer continues to rise, it is becoming increasingly crucial to diagnose it initially developed, research demonstrate that early identification and therapy improve the survival rate of skin cancer patients. With the advancement of medical research and AI, several skin cancer smartphone applications have been introduced to the market, allowing people with worrisome lesions to use a specialized technique to determine whether they should seek medical care. According to studies, over 235 dermatology smartphone apps were developed between 2014 and 2017 (Flaten et al. 2020). Previously, they worked by sending a snapshot of the lesion over the internet to a health care provider. Still, thanks to smartphones' internal AI algorithms, these applications can detect and classify images of lesions as high or low risk and Immediately assess the patient's risk and offer advice. SkinVison (Carvalho et al. 2019) is an example of a successful application.

## Future challenges

### Digital twinning in drug discovery

The development and implementation of Industry 4.0 emerging technologies allow for creation of digital twins (DTs), that promotes the modification of the industrial sector into a more agile and intelligent one. A DT is a digital depiction of a real entity that interacts in dynamic, two-way links with the original. Today, DTs are being used in a variety of industries. Even though the pharmaceutical sector has grown to accept digitization to embrace Industry 4.0, there is yet to be a comprehensive implementation of DT in pharmaceutical manufacture. As a result, it is vital to assess the pharmaceutical industry's success in applying DT solutions (Chen et al. 1088).

New digital technologies are essential in today's competitive marketplaces to promote innovation, increase efficiency, and increase profitability (Legner et al. 2017). AI (Venkatasubramanian 2019), Internet of Things (IoT) devices (Venkatasubramanian 2019; Oztemel and Gursev 2018), and DTs have all piqued the interest of governments, agencies, academic institutions, and corporations (Bao et al. 2018). Industry 4.0 is a concept offered by a professional community to increase the level of automation to boost productivity and efficiency in the workplace.

This section provides a quick look at the evolution of DT and its application in pharmaceutical and biopharmaceutical production. We begin with an overview of the technology's principles and a brief history, then present various examples of DTs in pharmacology and drug discovery. After then, there will be a discussion of the significant technical and other issues that arise in these kinds of applications.

#### History and main concepts of digital twin

The idea of making a "twin" of a process or a product returned to NASA's Apollo project in the late 1960s (Rosen et al. 2015; Mayani et al. 2018; Schleich et al. 2017), when it assembled two identical space spacecraft. In this scenario, the "twin" was employed to imitate the counterpart's action in real-time.

The DT, according to Guo et al. (2018), is a type of digital data structure that is generated as a separate entity and linked to the actual system. Michael Grieves presented the original meaning of a DT in 2002 at the University of Michigan as part of an industry presentation on product lifecycle management (PLM) (Grieves 2014; Grieves and Vickers 2017; Stark et al. 2019). However, the first actual use of this notion, which gave origin to the current moniker, occurred in 2010, when NASA (the United States National Aeronautics and Space Administration) attempted to create virtual spaceship simulators for testing (Glaessgen and Stargel 2012).

A digital reproduction or representation of a physical thing, process, or service is what a DT is in theory. It's a computer simulation with unique features that dynamically connect the physical and digital worlds. The purpose of DTs is to model, evaluate, and improve a physical object in virtual space til it matches predicted performance, at which time it can be created or enhanced (if already built) in the real world (Kamel et al. 2021; Marr 2017).

**Fig. 14:**
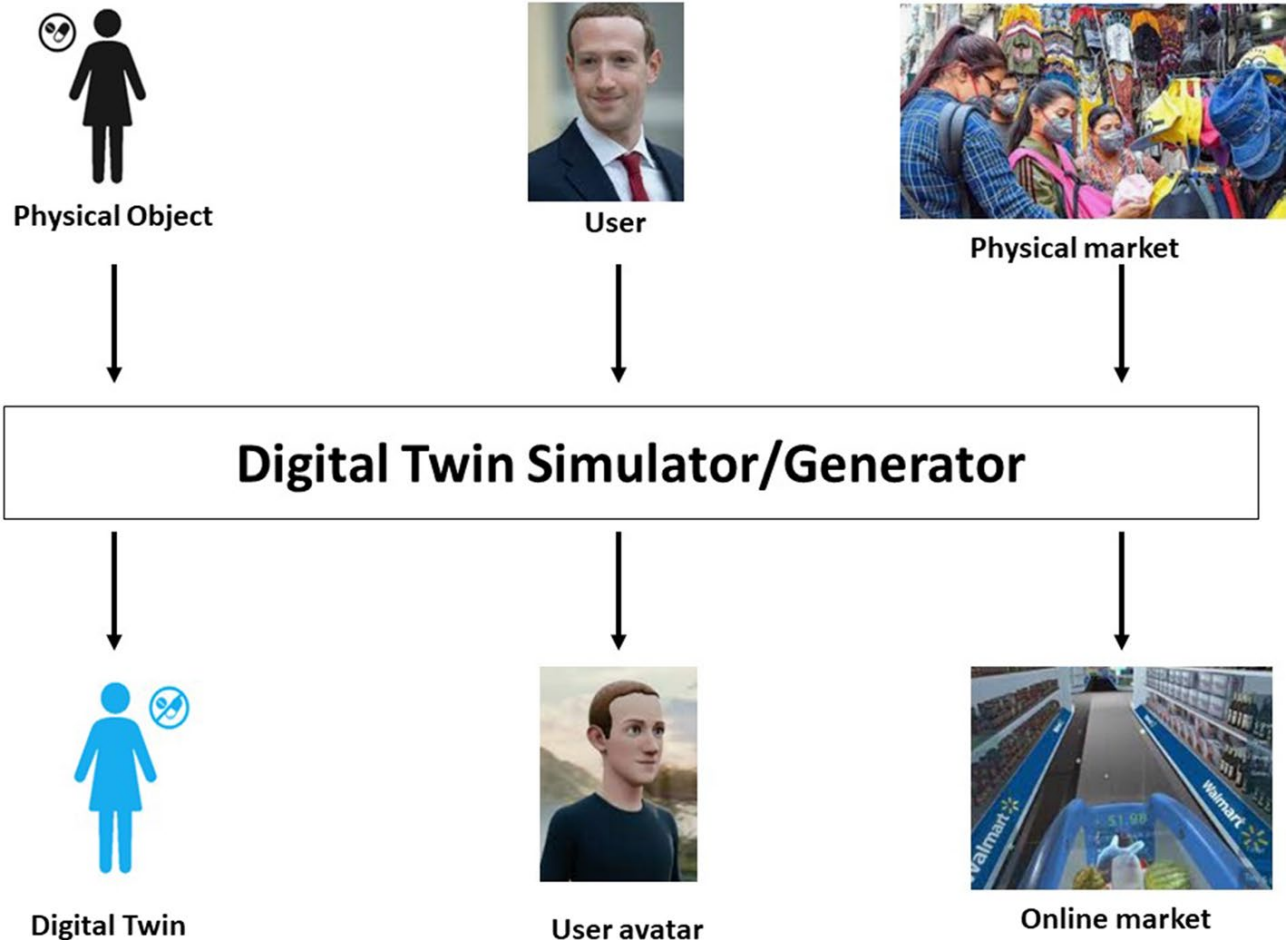
Main components of DT

Since then, DT technology has acquired popularity in both business and academia. Main components of DTs presently exist, as shown in Fig. 14. Still, the theoretical model comprises three parts: the real entity in the actual world, the digital entity in the virtual space, and the interconnection between them (Glaessgen and Stargel 2012).

In an ideal world, the digital component would have all the system's information that could be acquired from its physical counterpart (Kritzinger et al. 2018). When integrated with AI, IoT, and other recent intelligent systems, a DT can forecast how an object or process will perform.

#### Digital twin in pharmaceutical manufacturing

Developing a drug is lengthy and costly, requiring efforts in biology, chemistry, and manufacturing, and it has a low success rate. An estimated 50,000 hits (trial versions of compounds that are subsequently tweaked to develop a medication in the future) are evaluated to develop a successful drug. Only one in every 12 therapeutic compounds, clinical trials have been performed on humans, makes it to market successfully. Toxicity (A medication's capacity to offer a patient with respite and slow the progression of a disease) and lack of effectiveness contribute to more than 60% of all drug failures (Subramanian 2020).

Making the appropriate decisions about which targets, hits, leads, and compounds to pursue is important to a drug's successful market introduction. However, the decision is based on in vitro (Experimental system in a test tube or petri dish.) and in vivo (experiments in animals.) systems, both of which have a shaky correlation with clinical outcomes (Mak et al. 2014). Answers to the following inquiries would be provided by a perfect decision support system for drug discovery:

- What is the magnitude of any target's influence on the desired clinical result?
- Is the potential compound changing the target enough to change clinical outcomes?
- Is the chemical sufficiently selective and free of side effects or harmful consequences?
- Is the ineffectiveness attributable to the drug's failure to reach its target?
- Has the trial chosen the appropriate dose and dosing regimen?
- Are there any surrogate or biomarkers such as cholesterol that serves as a proxy for the illness's root cause that can forecast a drug's success or failure?
- Have the correct patients been chosen for the study?
- Is it possible to identify hyper- and hypo-responders before the study begins?

Therapeutic failures are prevalent and difficult to address, given the complex process of developing drugs based on the points above. This issue must be addressed by combining data and observations from many stages of the drug development process and developing a system that can forecast an experiment's outcome or a chemical modification's influence on a therapeutic molecule. This highlights the significance of DT in the field of drug discovery.

In the United States, funding organizations such as DARPA, NSF, and DOE have aggressively supported bioprocess modeling at the genomic and cellular levels, resulting in high-profile programs such as BioSPICE (Kumar and Feidler 2003). These groups have shown that smaller models built to answer specific issues can greatly influence drug development efficiency. This would make it possible to apply the prediction methodology to various stages of the drug discovery and research process, including confirmation of the target, enhancing leads, and choosing candidates, Recognition of biomarkers, fabrication of assays and screens, and the improvement of clinical trials.

The pharmaceutical business is embracing the overall digitization trend in tandem with the US FDA's ambition to establish an agile, adaptable pharmaceutical manufacturing sector that delivers high-quality pharmaceuticals without considerable regulatory scrutiny (O’Connor et al. 2016). Industries are beginning to implement Industry 4.0 and DT principles and use them for development and research (Barenji et al. 2019; Steinwandter et al. 2019; Lopes et al. 2019; Kumar et al. 2020; Reinhardt et al. 2020). Pharma 4.0 (Ierapetritou et al. 2016) is a digitalization initiative that integrates Industry 4.0 with International Council for Harmonisation (ICH) criteria to model a combined operational model and production control plan.

**Fig. 15:**
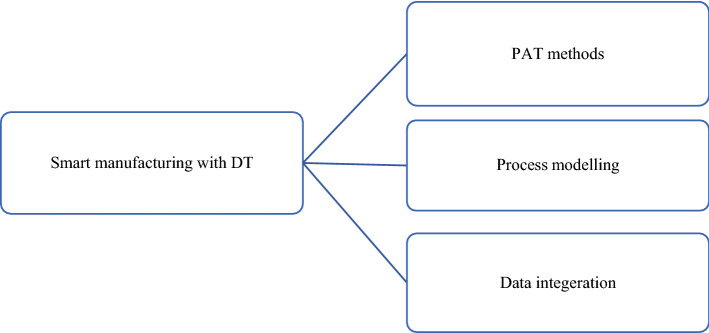
Main categories of smart manufacturing with DT

As shown in Fig. 15, live monitoring of the system `by the Process Analytical Technology (PAT), data collection from the machinery, the supplementary and finished goods, and a worldwide modelling and software for data analysis are some of the key requirements for achieving smart manufacturing with DT (Barenji et al. 2019). Quality-by-Design (QbD) and Continuous Manufacturing (CM) (Boukouvala et al. 2012), flowsheet modeling (Kamble et al. 2013), and PAT implementations (James et al. 2006) have all been used by the pharmaceutical industry to achieve this. Although some of the instruments have been thoroughly examined, DTs' entire integration and development is still a work in progress.

The pharmaceutical industry has used PAT in different programs across the steps involved in producing drugs (Nagy et al. 2013). Even though this has resulted in a rise in the use of PAT instruments, their implementations are limited to research and development rather than manufacturing on a large scale (Papadakis et al. 2018). They have been successful in decreasing production costs and enhancing product quality monitoring in the small number of examples where they have been used in manufacturing (Simon et al. 2019). The development of various PAT approaches, as well as their convincing implementation is a vital component of a scheme for surveillance and control (Boukouvala et al. 2012) and has given a foundation for obtaining essential data from the physical component.

Papadakis et al. (2018) recently provided a framework for identifying efficient reaction paths for pharmaceutical manufacture (Rantanen and Khinast 2015), which comprises modeling reaction route workflows discovery, analysis of reactions and separations, process simulation, assessment, optimization, and the use (Sajjia et al. 2017).

To develop models, data-driven modeling methods require the gathering and using of many substantial experiments, and the resulting models are solely reliant on the datasets provided. Artificial neural networks (ANN) (Pandey et al. 2006; Cao et al. 2018), multivariate statistical analysis, and in Monte Carlo Badr and Sugiyama (2020) are all commonly used in pharmaceutical manufacturing. These methods are less computationally costly, but the prediction outside the dataset space is frequently unsatisfactory due to the trained absence of underlying physics understanding in models. Using IoT devices in pharmaceutical manufacturing lines results in massive data collection volumes. The virtual component must receive this collection of process data and CQAs quickly and effectively. Additionally, for accurate prediction, several pharmaceutical process models need material properties. As a result, to provide virtual component access to all datasets, a central database site is necessary (Lin-Gibson and Srinivasan 2019).

#### Digital twin in biopharmaceutical manufacturing

The synthesis of big molecule-based entities in various combinations that has applications in the treatment of inflammatory, microbial, and cancer issues, is the focus of biopharmaceutical manufacturing (Glaessgen and Stargel 2012; Narayanan et al. 2020). The demand for biologic-based medications has risen in recent years, necessitating greater production efficiency and efficacy (Kamel et al. 2021). As a result, many businesses are switching from batch to continuous production and implementing intelligent manufacturing systems (Lin-Gibson and Srinivasan 2019). DT can aid in decision-making, risk analysis, product creation, and process prediction., which incorporates the physical plant, data collecting, data analysis, and system control (Tao et al. 2018).

biological products' components and structures are intimately connected to treatment effectiveness (Read et al. 2010) and are very sensitive to cell-line. Operating conditions thorough actual plant's virtual description in a simulation environment is required to apply DT in biopharmaceutical manufacturing (Tao et al. 2018). This means that each unit activity inside an integrated model's simulation should accurately reflect the crucial process dynamics. Previous reviews Narayanan et al. (2020) Tang et al. (2020) Farzan et al. (2017) Baumann and Hubbuch (2017) Smiatek et al. (2020) and Olughu et al. (2019) focused on process modelling methodologies for both upstream and downstream operations.

Data from a biopharmaceutical monitoring system is typically diverse regarding data kinds and time scales. A considerable amount of data is collected during biopharmaceutical manufacture thanks to the deployment of real-time PAT sensors. As a result, data pre-processing is required to deal with missing data, visualize data, and reduce dimensions (Gangadharan et al. 2019). In batch biopharmaceutical production, Casola et al. (2019) presented data mining-based techniques for stemming, classifying, filtering, and clustering historical real-time data. Lee et al. (2012) combined different spectroscopic techniques and used data fusion to forecast the composition of raw materials.

### AI-driven digital twins in today's pharmaceutical drug discovery

**Fig. 16:**
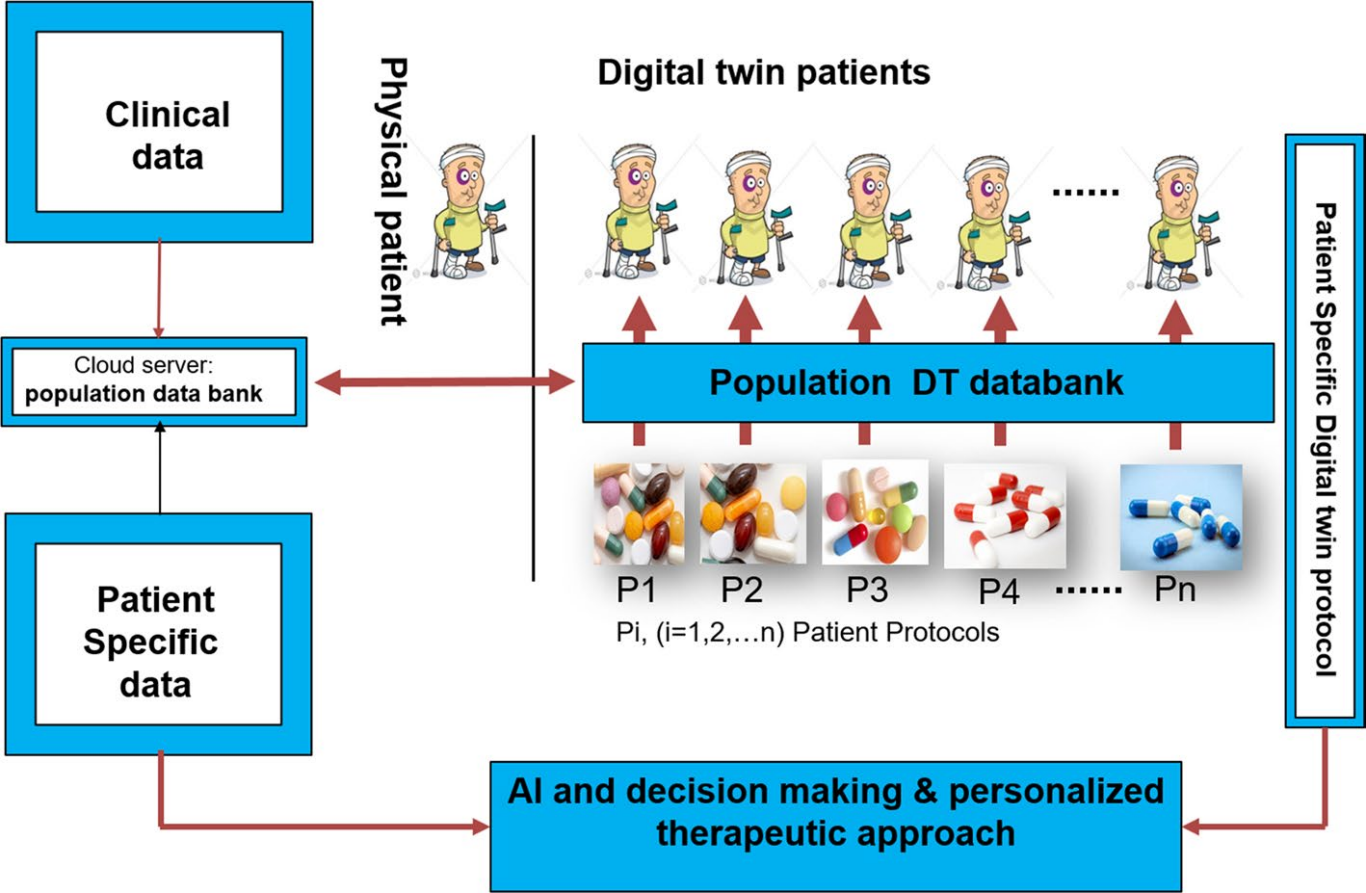
AI-driven digital twins in today's pharmaceutical drug discovery

In the pharmaceutical industry, challenges are emerging from clinical studies that make drug development incomplete, sluggish, uncertain, and maybe dangerous. For example, It is not a true reflection of reality where clinical trials can take into account that in the real world, just a small portion of a big and diverse population is depicted among the many billions of humans on the planet where it is not possible to get a view of how each person based on how they will respond to a medicine. Clinical trials' rigorous requirements for physical and mental health in some cases also result in failure because of a lack of qualified participants. Pharmaceutical firms battle to provide the precise number and kind of participants needed to comply with the stringent requirements of clinical trial designs. Also, in most trials, the actual drug is replaced by a placebo as this helps contrast how sick individuals behave when they are not administered the experimental medication; This implies that at least some trial participants do not receive it. Here, These issues can be solved by using digital twins, which can imitate a range of patient features, giving a fair representation of how a medicine affects a larger population. AI-enabled digital twinning may reduce the trial's setup by revealing how susceptible a patient is to various inclusion and exclusion criteria as a result, patients can be rapidly identified, and digital twins can predict a patient's reaction, and placebos won't be required. Therefore, the new treatment can be assured for every patient in the trial, and digital twins can reduce the dangerous impact of drugs in the early stages by decreasing the number of patients who need to be tested in the real world. Figure 16 illustrates a framework by running all possible combinations. All treatment protocols are tested on a digital twin of the patient to discover an appropriate treatment protocol for this patient. Doing this quickly and accurately can lead to providing the best quality treatment for the patient without experimenting with the patient, which saves effort, cost, and accuracy in determining an appropriate treatment protocol for patients.

## Open problems

This section discusses important issues to consider regarding progression from preclinical to clinical and implementation in practice that necessitate new ML solutions to assist transparent, usable, and data-driven decision-making procedures to accelerate drug discovery and decrease the number of failures in clinical development phases.

- Complex disorders, such as viral infections and advanced malignancies frequently necessitate drug combinations (Julkunen et al. 2020; White et al. 2021). For example, kinase inhibitor combos or single compounds that block several kinases may improve therapeutic efficacy and duration while combating treatment resistance in cancer (Attwood et al. 2021). While several ML models have been created to predict response pairs of drug–dose combinations, higher-order combination effects can be predicted in a systematic way involving more than two medicines or targets is still a problem. In cancer cell lines, tensor learning methods have permitted reliable prediction of paired drug combination dose-response matrices (Smiatek et al. 2020). This computationally efficient learning approach could use extensive pharmacogenomic data, determine which drug combinations are most successful for additional in vitro or in vivo testing in many kinds of preclinical models, such as higher-order combinations among novel therapeutic compounds and doses.
- While possible toxicity and effectiveness that is targeted are important criteria for clinical development success, most existing ML models for predicting response to the therapy accentuate effectiveness as the primary result. As a result, careful examination, and harmful effects prediction of instances in simulated and preclinical settings is required to strike a balance between the effectiveness of the toxicity and therapy that is acceptable to accelerate the next stages of drug development (Narayanan et al. 2020). Applying single-cell data and ML algorithms to develop combinations of anticancer drugs has shown the potential to boost the likelihood of clinical success (Tao et al. 2018). Transfer of knowledge and deconvolution techniques for in silico cell set (Avila et al. 2020) may offer effective ways to reduce the requirement to generate a lot of single-cell data to predict combination therapy responders and impacts of toxicity, as well as the recommended dosage that optimizes both efficacy and safety.
- In addition, patient data and clinical profiles must be used to validate the in-silico therapy response forecasts. This real data for ML predictions is crucial for progress in medicine and establishing the practical value and providing clinical guidance in making decisions. A no-go decision was made early, for example, if the substance has harmful consequences. Many of the present issues encountered when using machine learning for drug discovery, particularly in clinical development, are since current AI algorithms do not meet the requirements for clinical research. As a result, ML model validation requires systematic and comprehensive high-quality clinical data sets. The discovery methods must be thoroughly evaluated for accuracy and reproducibility using community-agreed performance measures in various settings, not just a small collection of exemplary data sets. sharing and exploiting private patient information is possible with systems that isolate the code from the data or use the model to data method (Guinney and Saez-Rodriguez 2018), which It makes it possible for federated learning to utilise patient-level data for model construction and thorough assessment.
- Even if there are many applications for drug discovery, The majority of ML and particularly DL models remain "black boxes”, and interpretation by a human specialist is sometimes tricky (Jiménez-Luna et al. 2020). Implementing mathematical models as online decision support tools must be understandable to users to obtain confidence. Comprehensible, accessible, and explainable models should clearly state the optimization goals, such as synergy, efficacy, and/or toxicity.
- DTI prediction is a notable example of fields of drug discovery research. It has been ongoing more than 10 years and aims to enhance the effectiveness of computational models using various technologies. The most recent computational approaches for predicting DTIs are DL technologies. These use unstructured-based approaches that don't need 3D structural data or docking to get over the drug and target protein's high-dimensional structure restrictions. Despite the DL's outstanding performance, regression inside the DTI prediction remains a critical and difficult issue, and researchers could develop several strategies to improve prediction accuracy. Furthermore, data scarcity and the lack of a standardized benchmark database are still considered current research gaps.
- While DL approaches show promise in detecting drug responses, especially when dealing with large amounts of data, drug response prediction research is in its first stages, and more efficient and relevant models are needed.
- While DL techniques have shown to be effective in detecting DDIs, especially when dealing with large amounts of data, more promising algorithms that focus on complex molecular reactions need to be developed.
- Only a few studies in the drug discovery field have investigated their models' explain ability, leaving much room for improvement. The explanations generated by XAI for human decision-making must be not insignificant, not artificial, and helpful to the scientific community. Until now, ensuring that XAI techniques achieve their goals and produce trustworthy responses would necessitate a combined effort amongst DL specialists, chemo informaticians and chemists, biologists, data scientists, and other subject matter experts. As a result, we believe that more developed methodologies to explain black-box models for drug discovery fields like DDIs, drug–target interactions, drug sensitivity, and drug side effects must be considered in the future to ensure model fairness or strict sensitivity evaluations of models. Further exploration of the capabilities and constraints of the existing chemical language for defining these models will be critical. The development of novel interpretable molecular representations for DL and the deployment of self-explanatory algorithms alongside sufficiently accurate predictions will be a critical area of research in the coming years. Because there are currently no methods that combine all the stated advantageous XAI characteristics (transparency, justification, informativeness, and uncertainty estimation), consensus techniques that draw on the advantages of many XAI approaches and boost model dependability will play a major role in the short and midterm. Currently, there is no open-community platform for exchanging and refining XAI software and model interpretations in drug discovery. As a result, we believe that future study into XAI in drug development has much potential.

## Discussion

This section presents a brief about how the proposed analytical questions in Sect. 2 are being answered through the paper.

- AQ1: What DL algorithms have been used to predict the different categories of drug discovery problems?
  Several DL algorithms have been used to predict the different categories of drug discovery problems as deeply illustrated in Sect. 4 with respect to the main categories of drug discovery problems in Fig. 8. In addition, a summary of a sample of these algorithms, their methods, advantages and weaknesses are presented in Table 2.
- AQ2: Which deep learning methods are mostly used in drug dosing optimization?
  Recognizing the characteristics that make medications suitable for precision dosage targets will aid in directing resources to where they'll have the most impact. Employing DL in drug dosing optimization is a big challenge which increases the health care performance, safety, and cost-effectiveness as presented in Sect. 7.
- AQ3: Are there any success stories about drug discovery and DL?
  With the advancement of DL methods, we've seen big pharmaceutical businesses migrate toward AI, such as ‘AstraZeneca’ which is a global multinational pharmaceutical business that has successfully used AI in every stage of drug development. Several success stories have been presented in Sect. 9.
- AQ4: What about using the newest technologies such as XAI and DT in drug discovery?
  The topic of XAI addresses one of the most serious flaws in ML and DL algorithms: model interpretability and explain ability. It would be impossible to trust the forecasts of real-world AI applications without interpretability and explain ability. Section 8 presents the literature that address this issue. A digital twin (DT) is a virtual representation of a living thing that is connected to the real thing in dynamic, reciprocal ways. Today, DTs are being used in a variety of industries. Even though the pharmaceutical sector has grown to accept digitization to embrace Industry 4.0, there is yet to be a comprehensive implementation of DT in pharmaceutical manufacture. Success stories regarding employing DT into drug discovery is presented in Sect. 10.
- AQ5: What are the future and open works related to the drug discovery and DL?.
  Through the paper, we present how DL succeed in all aspects of drug discovery problems, However, it is still a very important challenge for future research. Section 11 covers these challenges.

**Fig. 17:**
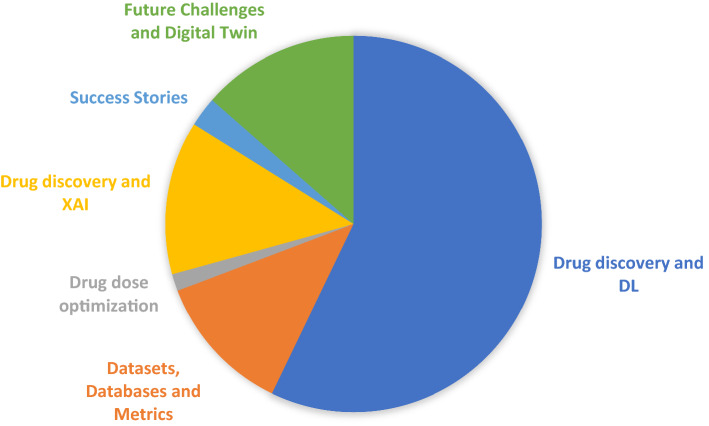
Percentages of DL applications for each category

Figure 17 presents the percentage of the different DL applications for each building block of our study. It is well observed that the most percentage segment is dedicated for the drug discovery and DL because it is the main core of our research.

## Conclusion

Despite all the breakthroughs in pharmacology, developing new drugs still requires a lot of time and costs. As DL technology advances and the amount of drug-related data grows, a slew of new DL-based approaches is cropping up at every stage of the drug development process. In addition, we’ve seen large pharmaceutical corporations migrate toward AI in the wake of the development of DL approaches.

Although the drug discovery is a large field and has different research categories, there is a few review studies about this field and each related study has focused only on a one research category such as reviewing the DL applications for the DTIs. So, the main goal of our research is to present a systematic Literature review (SLR) which integrates the recent DL technologies and applications for the different categories of drug discovery problems Including, Drug–target interactions (DTIs), drug–drug similarity interactions (DDIs), drug sensitivity and responsiveness, and drug-side effect predictions. That is associated with the benchmark data sets and databases. Related topics such as XAI and DT and how they support the drug discovery problems are also discussed. In addition, the drug dosing optimization and success stories are presented as well. Finally, we suggest open problems as future research challenges.

Although the DL has proved its strength in drug discovery problems, it is still a promising open research area for the interested researchers. In this paper, they can find all they want to know about using DL in various drug discovery problems. In addition, they can find success stories and open areas for future research.

Given the recent success of DL approaches and their use by pharmaceuticals in identifying new medications, it seems clear that current DL techniques being highly regarded in the next generation of enormous data investigation and evaluation for drug discovery and development.
